# TIM-1 serves as a receptor for Ebola virus in vivo, enhancing viremia and pathogenesis

**DOI:** 10.1371/journal.pntd.0006983

**Published:** 2019-06-26

**Authors:** Bethany Brunton, Kai Rogers, Elisabeth K. Phillips, Rachel B. Brouillette, Ruayda Bouls, Noah S. Butler, Wendy Maury

**Affiliations:** Department of Microbiology and Immunology, University of Iowa, Iowa City, Iowa, United States of America; NIAID Integrated Research Facility, UNITED STATES

## Abstract

**Background:**

T cell immunoglobulin mucin domain-1 (TIM-1) is a phosphatidylserine (PS) receptor, mediating filovirus entry into cells through interactions with PS on virions. TIM-1 expression has been implicated in Ebola virus (EBOV) pathogenesis; however, it remains unclear whether this is due to TIM-1 serving as a filovirus receptor in vivo or, as others have suggested, TIM-1 induces a cytokine storm elicited by T cell/virion interactions. Here, we use a BSL2 model virus that expresses EBOV glycoprotein to demonstrate the importance of TIM-1 as a virus receptor late during in vivo infection.

**Methodology/Principal findings:**

Infectious, GFP-expressing recombinant vesicular stomatitis virus encoding either full length EBOV glycoprotein (EBOV GP/rVSV) or mucin domain deleted EBOV glycoprotein (EBOV GPΔO/rVSV) was used to assess the role of TIM-1 during in vivo infection. GFP-expressing rVSV encoding its native glycoprotein G (G/rVSV) served as a control. TIM-1-sufficient or TIM-1-deficient BALB/c interferon α/β receptor^-/-^ mice were challenged with these viruses. While G/rVSV caused profound morbidity and mortality in both mouse strains, TIM-1-deficient mice had significantly better survival than TIM-1-expressing mice following EBOV GP/rVSV or EBOV GPΔO/rVSV challenge. EBOV GP/rVSV or EBOV GPΔO/rVSV in spleen of infected animals was high and unaffected by expression of TIM-1. However, infectious virus in serum, liver, kidney and adrenal gland was reduced late in infection in the TIM-1-deficient mice, suggesting that virus entry via this receptor contributes to virus load. Consistent with higher virus loads, proinflammatory chemokines trended higher in organs from infected TIM-1-sufficient mice compared to the TIM-1-deficient mice, but proinflammatory cytokines were more modestly affected. To assess the role of T cells in EBOV GP/rVSV pathogenesis, T cells were depleted in TIM-1-sufficient and -deficient mice and the mice were challenged with virus. Depletion of T cells did not alter the pathogenic consequences of virus infection.

**Conclusions:**

Our studies provide evidence that at late times during EBOV GP/rVSV infection, TIM-1 increased virus load and associated mortality, consistent with an important role of this receptor in virus entry. This work suggests that inhibitors which block TIM-1/virus interaction may serve as effective antivirals, reducing virus load at late times during EBOV infection.

## Author summary

T cell immunoglobulin mucin domain-1 (TIM-1) is one of a number of phosphatidylserine (PS) receptors that mediate clearance of apoptotic bodies by binding PS on the surface of dead or dying cells. Enveloped viruses mimic apoptotic bodies by exposing PS on the outer leaflet of the viral membrane. While TIM-1 has been shown to serve as an adherence factor/receptor for filoviruses in tissue culture, limited studies have investigated the role of TIM-1 as a receptor in vivo. Here, we sought to determine if TIM-1 was critical for Ebola virus glycoprotein-mediated infection using a BSL2 model virus. We demonstrate that loss of TIM-1 expression results in decreased virus load late during infection and significantly reduced virus-elicited mortality. These findings provide evidence that TIM-1 serves as an important receptor for Ebola virus in vivo. Blocking TIM-1/EBOV interactions may be effective antiviral strategy to reduce viral load and pathogenicity at late times of EBOV infection.

## Introduction

*Zaire ebolavirus* (EBOV) is one of five species of ebolaviruses within the *Filoviridae* family. EBOV continues to cause significant outbreaks in sub-Saharan Africa with case fatality rates as high as 90% [1]. All filoviruses have a broad species and cellular tropism. With the exception of lymphocytes, most cells within the body are thought to support EBOV infection and replication [2, 3]. Histopathological studies of EBOV infected humans and non-human primates (NHPs) have demonstrated viral antigen in many different organs including: liver, spleen, lymph nodes, kidney, adrenal glands, lungs, gastrointestinal tract, skin, brain and heart [3–7].

Numerous cell surface receptors are appreciated to mediate filovirus binding and internalization into the endosomal compartment of cells, including phosphatidylserine (PS) receptors [8, 9] and C-type lectin receptors [10–14]. PS receptors do not interact with the viral glycoprotein (GP), but bind to PS on the surface of the virion lipid membrane, causing internalization of viral particles into the endosomal compartment [9, 15]. This mechanism of viral entry has been termed apoptotic mimicry [16]. Following endosomal uptake of filovirions, proteolytic GP processing occurs, thereby allowing GP to interact with its endosomal cognate receptor, Niemann Pick C1 [17–21].

One important family of PS receptors is the T-cell immunoglobulin mucin domain (TIM) family. TIM family members, encoded by the *Havcr* family of genes, contribute to the uptake of apoptotic bodies to clear dying cells from tissues and the circulation [22–24]. TIM proteins are type 1, cell surface glycoproteins. Three family members are present in humans (hTIM-1, hTIM-3 and hTIM-4) and four in mice (TIM-1, TIM-2, TIM-3 and TIM-4) [25]. hTIM-1 was identified through a bioinformatics-based screen to be important for filovirus entry [8]. Subsequent studies demonstrated that hTIM-1 and hTIM-4, but not hTIM-3, enhance entry of a broad range of viruses including members of the alphavirus, arenavirus, baculovirus, filovirus, and flavivirus families [9, 15, 26–29]. Murine TIM-1 and TIM-4 also enhance enveloped virus uptake into the endosomal compartment [9, 27, 29].

The molecular interactions between TIM family members and enveloped viruses are well defined. The amino terminal IgV domain binds to PS on the outer leaflet of the viral membrane through a IgV domain binding pocket that is conserved across the TIM family of receptors [9, 26, 27, 29]. We have reported that the ability of PS on Ebola virus like particles and EBOV glycoprotein pseudotyped vesicular stomatitis virus to bind to TIM-1 is equivalent, suggesting similar levels of PS present on the surface of these virions [15]. Aspartic acid and asparagine residues within the IgV binding pocket are essential for virion binding [9, 15, 27]; these same TIM residues are required for apoptotic body binding and uptake [30]. The IgV domain is extended from the plasma membrane by a mucin like domain (MLD) that is anchored to the cell surface with by a transmembrane domain connected to a short intracellular cytoplasmic tail. The length, but not the specific sequence, of the MLD is required for TIMs to serve as enveloped virus receptors [29]. Surprisingly, neither the TIM transmembrane domain nor cytoplasmic tail is required as a GPI-anchored TIM-1 construct is completely functional as a viral receptor [26, 29]. These findings indicate that the TIM-1 cytoplasmic tail, which contains a tyrosine phosphorylation site that initiates signaling events [31–33], is not essential for TIM-1-mediated virus uptake.

While it is well established that TIM proteins serve as cell surface receptors for a number of enveloped viruses during infection of cultured cells, the importance of these family members for in vivo filovirus infection and pathogenesis has not been extensively examined. With the wide variety of cell surface receptors able to mediate filovirus uptake into endosomes, it is possible that sufficient receptor redundancy exists in vivo, such that the loss of any one of the PS receptors may have little or no effect on EBOV viremia, tissue virus load or pathological consequence. Alternatively, specific cell surface receptors, such as TIM-1, might be critical for in vivo infection and pathogenesis.

As PS receptors have been reported to mediate both immunomodulatory and proinflammatory responses [34–37], an additional impact of TIM proteins on virus infection may be due to alterations in innate immune responses. A recent study demonstrated that TIM-1-deficient mice have lower morbidity and mortality than wild-type mice when challenged intravascularly (i.v.) with mouse-adapted EBOV (maEBOV) [38]. This study highlighted the role of TIM-1 in non-permissive T lymphocytes, reporting that EBOV interaction with TIM-1 on CD4+ T cells enhanced proinflammatory cytokine dysregulation in purified CD4^+^ T cells. The authors conclude that an enhanced TIM-1-dependent cytokine storm in T cells significantly contributes to EBOV pathogenesis. However, the impact of TIM-1 on viremia in mice was examined in the plasma at a single time point during infection, leaving open the possibility that TIM-1 may also serve as an important receptor for EBOV entry in vivo.

Here, we examined the in vivo importance of TIM-1 for virus replication and pathogenesis using a highly tractable BSL2 model virus of EBOV. Our BSL2 virus model is recombinant vesicular stomatitis virus (VSV) encoding either full length EBOV glycoprotein or mucin domain deleted EBOV glycoprotein in place of the native VSV G protein (EBOV GP/rVSV or EBOV GPΔO/rVSV). Our use of these viruses allowed us to conduct detailed studies focused, on the role of TIM-1 virus entry, host responses, and pathogenesis. As reported for maEBOV, we observed that both EBOV GP/rVSV and EBOV GPΔO/rVSV were less pathogenic in TIM-1-deficient mice compared to TIM-1-sufficient mice. The impact of the loss of TIM-1 was specific for EBOV GP-expressing viruses since wild-type VSV was equally virulent in TIM-1-deficient and TIM-1-sufficient mice over a wide range of challenge doses. Importantly, reduced mortality observed in the EBOV GP encoding virus-infected TIM-1^-/-^ mice was associated at late times during infection with lower viremia and virus loads in multiple tissues previously appreciated to be important in EBOV pathogenesis. Consistent with reduced overall virus loads, proinflammatory chemokine profiles were lower in the infected TIM-1-deficient mice at late times during infection. Finally, to directly evaluate whether we observed enhanced pathogenesis in TIM-1-sufficient mice associated with T cell activation as previously reported [38], we depleted the T cell compartment of TIM-1-sufficient or -deficient mice and challenge them with EBOV GP/rVSV. T cell-depleted, TIM-1-sufficient mice succumbed to EBOV GP/rVSV more readily than T cell-depleted, TIM-1-deficient mice, suggesting that in our model system a TIM-1-dependent T cell cytokine storm was not responsible for virus pathogenesis. In total, our studies provide evidence that TIM-1-associated pathogenesis correlated with enhanced virus load at late times during infection, consistent with TIM-1 having an important role as a receptor for EBOV in vivo.

## Materials and methods

### Ethics statement

This study was conducted in strict accordance with the Animal Welfare Act and the recommendations in the Guide for the Care and Use of Laboratory Animals of the National Institutes of Health (University of Iowa (UI) Institutional Assurance Number: #A3021-01). All animal procedures were approved by the UI Institutional Animal Care and Use Committee (IACUC) which oversees the administration of the IACUC protocols and the study was performed in accordance with the IACUC guidelines (Protocol #8011280, Filovirus glycoprotein/cellular protein interactions).

### Mice

BALB/c TIM-1-deficient mice have been previously described [39] and were a kind gift from Dr. Paul Rothman (Johns Hopkins University). Briefly, exons 4 and 5 of the TIM-1 gene, *Havcr1*, were replaced with a LacZ gene, generating a TIM-1-null mouse (TIM-1^-/-^). BALB/c IFN-αβ receptor-deficient (*Ifnar*^-/-^) mice were a kind gift from Dr. Joan Durbin, NYU Langone Medical Center. Mice were bred at the University of Iowa.

BALB/c *Ifnar*^-/-^ and BALB/c *Havcr1*^-/-^ (TIM-1^-/-^) mice were crossed for the creation of heterozygous progeny. Progeny were interbred and mice screened for the correct BALB/c *Ifnar*^-/-^/*Havcr1*^-/-^ genotype (referred to as TIM-1^-/-^ throughout this study). Genomic DNA from mouse tail-clips was assessed by PCR for genotypes. All expected genotypes were produced in normal Mendelian ratios. The primers and protocol for *Ifnar*^-/-^ genotyping has been previously described [40]. *Havcr1* primer sequences included: shared forward, 5' GTTTGCTGCCTTATTTGTGTCTGG 3'; WT reverse, 5' CAGACATCA-ACTCTACAAGGTCCAAGAC 3'; knockout reverse, 5' GTCTGTCCTAGCTTCCTCACTG 3'. PCR amplification was performed for 30 cycles at 94°C for 30 sec, 60°C for 30 sec, and 72°C for 1 min.

### Production of full length EBOV GP/rVSV virus and EBOV GPΔO/rVSV which lacked the mucin-like domain

These studies used recombinant, replication-competent vesicular stomatitis virus (VSV) expressing GFP and either full length EBOV GP (EBOV GP/rVSV-GFP) [41] (kind gift of Dr. Kartik Chandran), EBOV GP lacking the mucin domain of GP1 (EBOV GPΔO/rVSV-GFP) [8, 15] or rVSV-GFP encoding its native glycoprotein, G (G/rVSV) (kind gift of Dr. Sean Whelan). Virus stocks were produced by infecting Vero cells, an African green monkey kidney epithelial cell line, at a low multiplicity of infection (MOI) of ~0.001 and collecting supernatants 48 hours following infection. Virus stocks were concentrated by centrifugation at 7,000 rpm at 4°C overnight. The virus pellet was resuspended and centrifuged through a 20% sucrose cushion by ultracentrifugation at 26,000 rpm for 2 hours at 4°C in a Beckman Coulter SW32Ti rotor. The pellet was resuspended in PBS, treated with endotoxin removal agent (ThermoScientific #20339), aliquoted, and frozen at -80°C until use.

### Mouse infections

Five- to eight-week-old female BALB/c *Ifnar*^-/-^ (control) and BALB/c *Ifnar*^-/-^*/Havcr1*^-/-^ (TIM-1^-/-^) mice were infected i.v. with recombinant, infectious VSV that encoded GFP and EBOV GP, EBOV ΔO or the native VSV G glycoprotein (EBOV GP/rVSV-GFP, EBOV GPΔO/rVSV-GFP and G/rVSV-GFP, respectively) using concentrations of virus noted in the figure legends. The dose of EBOV GP/rVSV or EBOV GPΔO/rVSV-GFP administered was dependent upon the stock. The dose of each stock was titered in vivo to identify stock concentrations that gave predictably high (75% or greater of challenged mice) levels of mortality of *Ifnar*^*-/-*^ (control) mice in 5–7 days. For studies with G/rVSV-GFP, either 10^1^ or 10^5^ iu of VSV virus was administered by i.v. injection. Survival was tracked; mice were weighed and scored for sickness daily. Clinical assessment of sickness was scored as follows: 0, no apparent illness; 1, slightly ruffled fur; 2, ruffled fur, active; 3, ruffled fur, inactive; 4, ruffled fur, inactive, hunched posture; 5, moribund or dead. Mice were humanely euthanized if they reached a score of 4. All mouse infection studies were concluded at 10 or 12 days following infection due to surviving mice regaining any lost weight and having no signs of clinical illness.

### Organ viral titers

Organs were harvested from control and TIM-1^-/-^ mice at 1, 3 or 5 days following infection from with EBOV GPΔO/rVSV. Prior to euthanasia, mice were anesthetized with isoflurane to perform retro-orbital bleeds for serum. Mice were euthanized and perfused with 10 mL of PBS through the heart and organs harvested, weighed and frozen at -80°C. To determine virus titers, organs or sera were thawed and organs were homogenized in PBS and filtered through a 0.45 μm syringe filter. Viral titers were determined by end-point dilution on Vero cell as previously described [8]. Infection was scored 5 days following infection for GFP positivity using an inverted fluorescent microscope. Virus titers were calculated as 50% tissue culture infective dose (TCID_50_)/mL by the Spearman-Karber method. All organ titers were normalized according to the weight of the organ at harvest.

### Organ RNA isolation and reverse transcriptase quantitative PCR

Quantitative reverse transcriptase polymerase chain reaction (qRT-PCR) was used to detect proinflammatory cytokine and chemokines levels from organs of mice challenged with EBOV GPΔO/rVSV. At time of harvest, organs were placed in Trizol and frozen at -80°C until further use. Total RNA was isolated using TRIzol LS reagent (Life Technologies) according to manufacturer’s tissue RNA isolation procedure. RNA was quantified by Nanodrop (Thermo Scientific). Total RNA (2 μg) was reverse transcribed into cDNA using random primers and the High-Capacity cDNA Reverse Transcription kit (Applied Biosystems). SYBR Green based quantitative PCR reactions (Applied Biosystems) were performed using 1.5μL of a 1:100 dilution of cDNA from each reaction and specific primers for murine cytokines and chemokines. Primer sequences are found in S1 Table. Expression levels of the cytokine/ chemokines of interest were defined as a ratio between threshold cycle (Ct) values for the gene of interest and the endogenous control, mouse hypoxanthine guanine phosphoribosyl transferase (HPRT), and is displayed as the log2 value of this ratio.

### T cell depletion studies

Five- to eight-week-old female BALB/c *Ifnar*^-/-^ and BALB/c *Ifnar*^-/-^/TIM-1^-/-^ mice were injected with 200μg of anti-CD4 (clone GK1.5) and 200μg anti-CD8 (clone 2.43) depleting monoclonal antibodies both one day prior to retro-orbital infection with EBOV GP/rVSV-GFP and two days post infection. Survival was tracked; mice were weighed and scored for sickness daily as described above to assess euthanasia criteria for each infected mouse. Prior to infection with EBOV GP/rVSV-GFP, depletion was validated by isolating peripheral blood mononuclear cells from both depleted and non-depleted animals and staining of PBMCs with anti-CD90 antibody (clone 30-H12). Staining was done by incubating with anti-CD90 antibody in FACS buffer and Fc block (clone 2.4G2) for 30 minutes, washing 3 times to remove excess antibody, and detecting fluorescence on a BD FACSCalibur.

### Statistics

Statistical analyses were performed using GraphPad Prism software (GraphPad Software, Inc.). Results are shown as means or geometric means and standard error of the means (s.e.m.) or geometric s.e.m., respectively, is shown where appropriate. Log-rank (Mantel-Cox) tests were used to analyze differences in survival. In vivo experiments were performed at least in duplicate with at least 8 mice total per treatment group. Mice or samples were randomly assigned to various treatment groups. All data points and animals were reported in results and statistical analyses. For the nonparametric viral titer data, Mann-Whitney U-test was used. *P* values less than 0.05 were considered significant. For two way comparisons between control and experimental values, a Student’s t-test was performed.

## Results

### TIM-1 enhances EBOV GP/rVSV or EBOV GPΔO/rVSV infection, but not VSV

To create a TIM-1 deficient mouse, exons 4 and 5 of the *Havcr1* gene encoding TIM-1 were replaced with the LacZ gene by homologous recombination as previously described [39]. This mouse strain was used to study the role of TIM-1 in allergic airway diseases and Th2 responses [39]. Phenotypic characterization of TIM-1^-/-^ mice revealed no differences in immune cell numbers, immune system development, or immunological homeostasis compared to WT mice [39]. BALB/c TIM-1^-/-^ mice were bred onto a BALB/c interferon αβ receptor (*Ifnar*^*-/-*^) knock out background since type I interferon abrogates replication of the BSL2 recombinant EBOV GP/rVSV used in these studies [41, 42]. Homozygous BALB/c *Ifnar*^-/-^/TIM-1^-/-^ and *Ifnar*^*-/-*^ mice (called TIM-1^-/-^ and control mice, respectively, throughout the remainder of this study) were used for all infections. Challenge virus was administered intravenously to mimic a primary route of EBOV transmission, blood-to-blood contact. Mice were challenged with the lowest dose of virus that produced predictable death in control mice in 5–7 days (S1 Fig). Minor titer variations were observed between virus stocks and dosages were adjusted accordingly.

**Fig 1:**
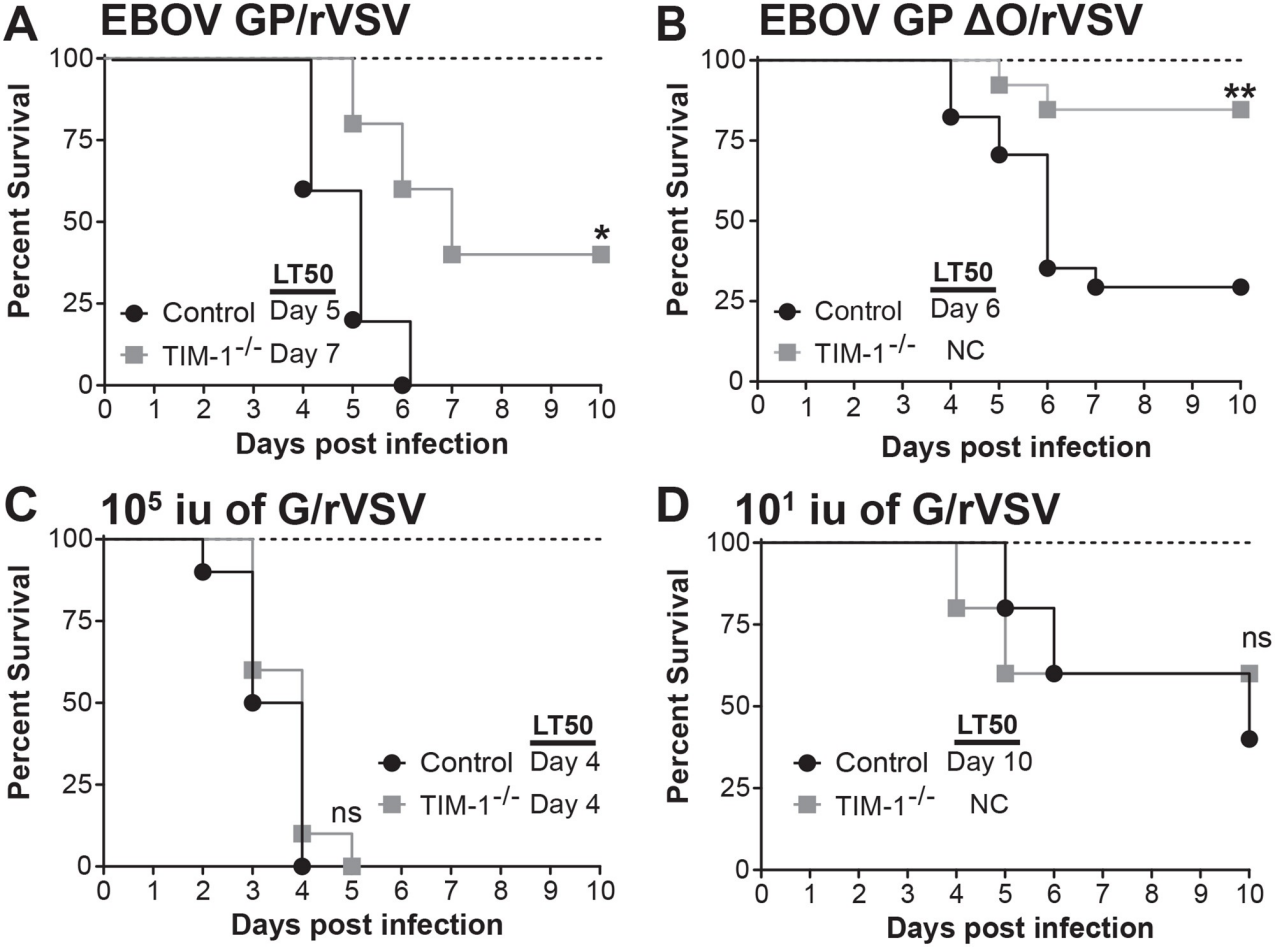
Loss of TIM-1 reduces mortality following EBOV GP/rVSV and EBOV GP ΔO/rVSV infection, but not G/rVSV. A and B. Female BALB/c *Ifnar*^-/-^ (control) and BALB/c *Ifnar* ^-/-^/TIM-1^-/-^ (TIM-1^-/-^) mice infected with 10^5^ iu EBOV GP/rVSV (A; n = 5 mice per group) or EBOV GP ΔO/rVSV (B; n = 13–17 mice per group) by intravenous (i.v.) injection. C. Female BALB/c *Ifnar* ^-/-^ (control) and BALB/c *Ifnar* ^-/-^/TIM-1^-/-^ (TIM-1^-/-^) mice infected with 10^5^ iu G/rVSV (n = 10 mice per group) by i.v. infection. D. Similar G/rVSV challenge studies as shown in panel C, but mice were challenged with 10^1^ iu (n = 5 mice per group) of G/rVSV. Survival was assessed following infection for all mouse studies. Significance for survival curve was determined by Log Rank (Mantel-Cox) test, * p< 0.05, **p < 0.01. LT50 = median lethal time until death; NC, noncalculable; ns, not significant.

We challenged the TIM-1^-/-^ and control mice with full length EBOV GP/rVSV or EBOV GPΔO/rVSV, which has the GP1 mucin like domain (MLD) deleted. EBOV GPΔO pseudovirions and recombinant viruses have the same tropism as virus bearing EBOV GP [8, 43–45]. Use of both viruses in these studies allowed us to determine if the elimination of the mucin domain altered the pathogenesis associated with in vivo challenge with these viruses. As expected, TIM-1-sufficient control mice succumbed to EBOV GP/rVSV or EBOV GPΔO/rVSV between days 4–7 of infection (Fig 1A and 1B). By contrast, TIM-1^-/-^ mice challenged with the same dose had significantly reduced mortality following EBOV GP/rVSV or EBOV GPΔO/rVSV infection and delayed time-to-death of those that did succumb to infection. These findings indicate that TIM-1^-/-^ mice had improved survival when infected with EBOV GP/rVSV compared to controls and that survival was not affected by the presence of the GP1 MLD.

In tissue culture studies, we have shown that hTIM-1 does not mediate WT VSV entry [8], presumably because the cognate receptor for VSV, LDL receptor, is abundantly present on target cells and mediates VSV entry [46]. However, the relevance of TIM-1 in vivo for VSV infection has not been examined. Further, WT VSV serves as an excellent control for in vivo studies with EBOV GP-bearing viruses. We challenged TIM-1^-/-^ and control mice with 10^5^ iu of VSV by i.v. injection. In contrast to our EBOV GP/rVSV findings, we observed no difference in the survival curve between the two strains of mice (Fig 1C). Since it is likely that VSV bearing it native GP is more pathogenic than a recombinant VSV containing a different viral GP, we also evaluated mortality associated with different doses of VSV and found that administration of as little as 10^1^ iu of VSV was lethal to *Ifnar*^-/-^ mice (S2 Fig). Thus, we repeated VSV infections in control and TIM-1^-/-^ mice at a challenge dose of 10^1^ iu to determine if subtle changes in virus pathogenesis could be discerned. Even at this low dose, there was no difference in the survival in the TIM-1^-/-^ mice versus the control mice (Fig 1D). These results provide evidence that the difference in EBOV GP/rVSV pathogenesis in BALB/c *Ifnar*^-/-^ and TIM-1^-/-^ mice was due to the presence of EBOV GP expressed in the recombinant VSV rather than other VSV genes. The reduced pathogenesis of EBOV GP expressing virus in TIM-1^-/-^ mice was consistent with findings described by Younan et al. using maEBOV [38].

### Murine TIM-1 enhances EBOV GPΔO/rVSV load at late times during infection

**Fig 2:**
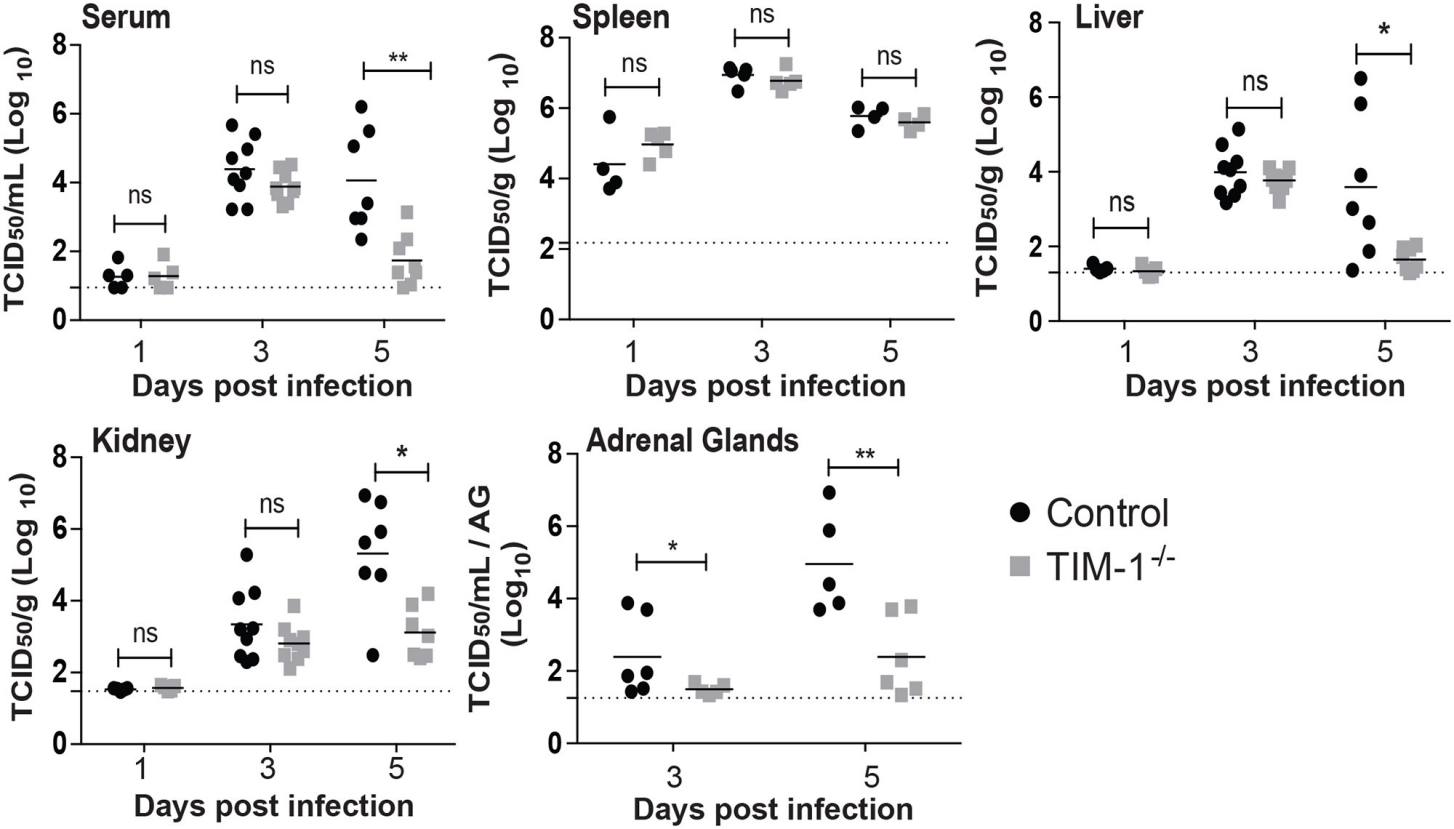
Reduced viremia and virus titers in a variety of organs of TIM-1^-/-^ mice at late time points following i.v. EBOV GP ΔO/rVSV infection. Serum and organs were harvested from BALB/c *Ifnar* ^-/-^ (Control) and BALB/c *Ifnar* ^-/-^/TIM-1^-/-^ (TIM-1^-/-^) mice at days 1, 3 and 5 following infection with 10^5^ iu of EBOV GP ΔO /rVSV by i.v. injection. Titers were determined by endpoint dilution of serum or homogenized organ samples on Vero cells. Solid lines indicate geometric mean for each data set. Dotted line indicates the level of detection. Adrenal gland titers are displayed as per gland homogenized in 1 ml of PBS. Significance was calculated by Mann-Whitney test to compare control to TIM-1^-/-^ mice at each time point; *p < 0.05; **p < 0.01; ns, not significant.

The effect of TIM-1 expression on viremia and organ viral loads following i.v. EBOV GPΔO/rVSV infection was examined in serum and organs harvested 1, 3 or 5 days following infection (Fig 2). Viremia and infectious virus in various organs were quantified by endpoint dilution titering on Vero cells, a highly permissive cell line for EBOV GPΔO/rVSV. At early times during infection, no difference in viremia or virus load was observed in most organs of TIM-1^-/-^ versus control mice. However, by day 5 of EBOV GPΔO/rVSV infection, TIM-1^-/-^ mice had a 100-fold reduction in viremia compared to control mice (Fig 2) and a similar trend was observed during infections with full length EBOV GP/rVSV (S3 Fig). In parallel, levels of infectious virus in liver, kidney, and adrenal gland were also significantly reduced. Studies at day 5 of infection also indicated that EBOV GPΔO/rVSV loads were much reduced in the brain of TIM-1^-/-^ mice and trended lower in the testis (S4A and S4B Fig), consistent with an overall reduction in virus load in the TIM-1^-/-^ mice at late times during infection. Thus, reduced virus replication in a number of organs was associated with the survival observed in TIM-1^-/-^ mice. These findings provide evidence that TIM-1 expression is important for the generation of high viral load in some organs at late times in infection.

Viral loads in the spleen and lungs were not affected by the loss of TIM-1 (Fig 2 and S4C Fig). The viral burden in the spleen was significantly higher at day 1 than in any other organ assessed and remained high in both mouse strains throughout the course of infection with a peak in titers occurring at day 3. These results are consistent with previous studies that implicate spleen in early and sustained EBOV replication [47–49]. Lung titers were not significantly different between the control and TIM-1^-/-^ mice at 5 days following infection. This result was somewhat unexpected as we had previously demonstrated robust hTIM-1 expression on the apical surface airway epithelial cells [8]. As TIM-1 was not observed to be expressed on the basolateral side of lung epithelium, TIM-1 may be important for entry of aerosolized EBOV entry into a host, but may not influence basolateral infection of lung via the circulation.

### TIM-1-expressing mice exhibit elevated levels of specific proinflammatory chemokines following EBOV GPΔO/rVSV infection

**Fig 3:**
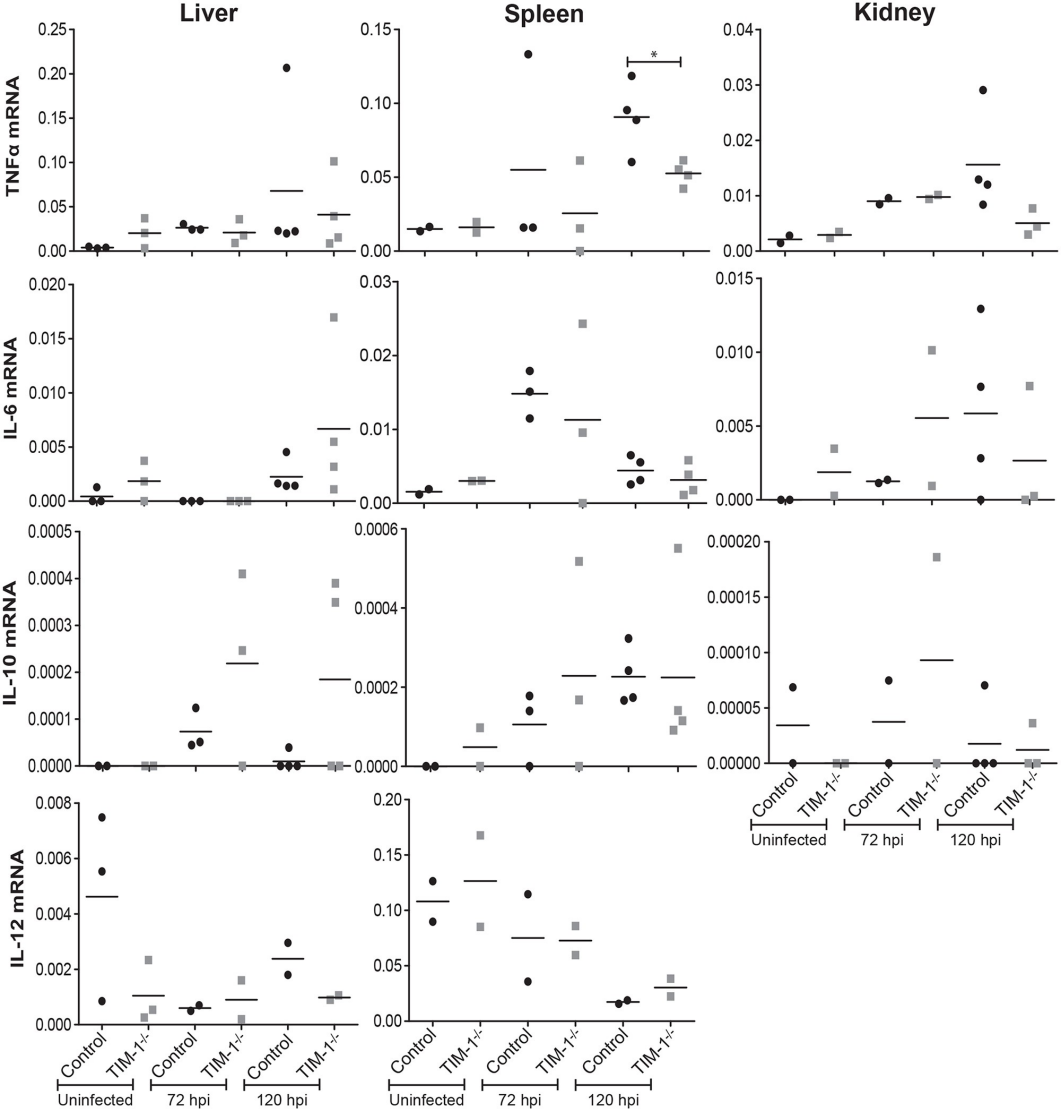
Cytokine expression in liver, spleen and kidney of EBOV GPΔO/rVSV-infected *Ifnar*^*-/-*^ and *Ifnar* ^-/-^/TIM-1^-/-^ mice. Tissues were harvested from uninfected and infected BALB/c *Ifnar* ^-/-^ (control) and BALB/c *Ifnar* ^-/-^/TIM-1^-/-^ (TIM-1^-/-^) mice. In infected mice, tissues were harvested at 3 or 5 days following infection with 10^5^ iu of EBOV GPΔO/rVSV by i.v. injection. RNA was isolated from the organs and expression of mouse TNF, IL-6, IL-10 and IL-12, was quantified by qRT-PCR. Results represent cytokines expression relative to murine HPRT for at least three independent livers, spleens and kidneys. Data points represent values for individual mice. Solid lines indicate the mean for each data set. Statistical significance was determined by Student’s t-test compared the control mice for each time point and is only shown for those comparisons observed to differ. *p<0.05.

Elevated proinflammatory and immunomodulatory cytokines and chemokines are evident in serum and infected organs during EBOV infection of animal models and patients [50–56]. To determine if reduced virus load in TIM-1-deficient mice at late time points was associated with lower RNA expression profiles of selected, well-characterized cytokines, levels in the spleen, liver and kidney were examined prior to and following EBOV GPΔO/rVSV infection. Organs were harvested at day 3 and 5 of infection and total RNA was isolated and amplified for the mRNA of the housekeeping gene, HPRT, and the cytokines TNF, IL-6, IL-12 and IL-10. Cytokine expression levels were normalized against mouse HPRT expression. Overall, baseline values of the organ cytokine expression from uninfected control and TIM-1^-/-^ mice were similar (Fig 3). While at day 5 of infection TNF was significantly higher in spleen of control mice, in general during infection the expression of cytokine was variable within groups and levels were not significantly different between the two strains of mice.

**Fig 4:**
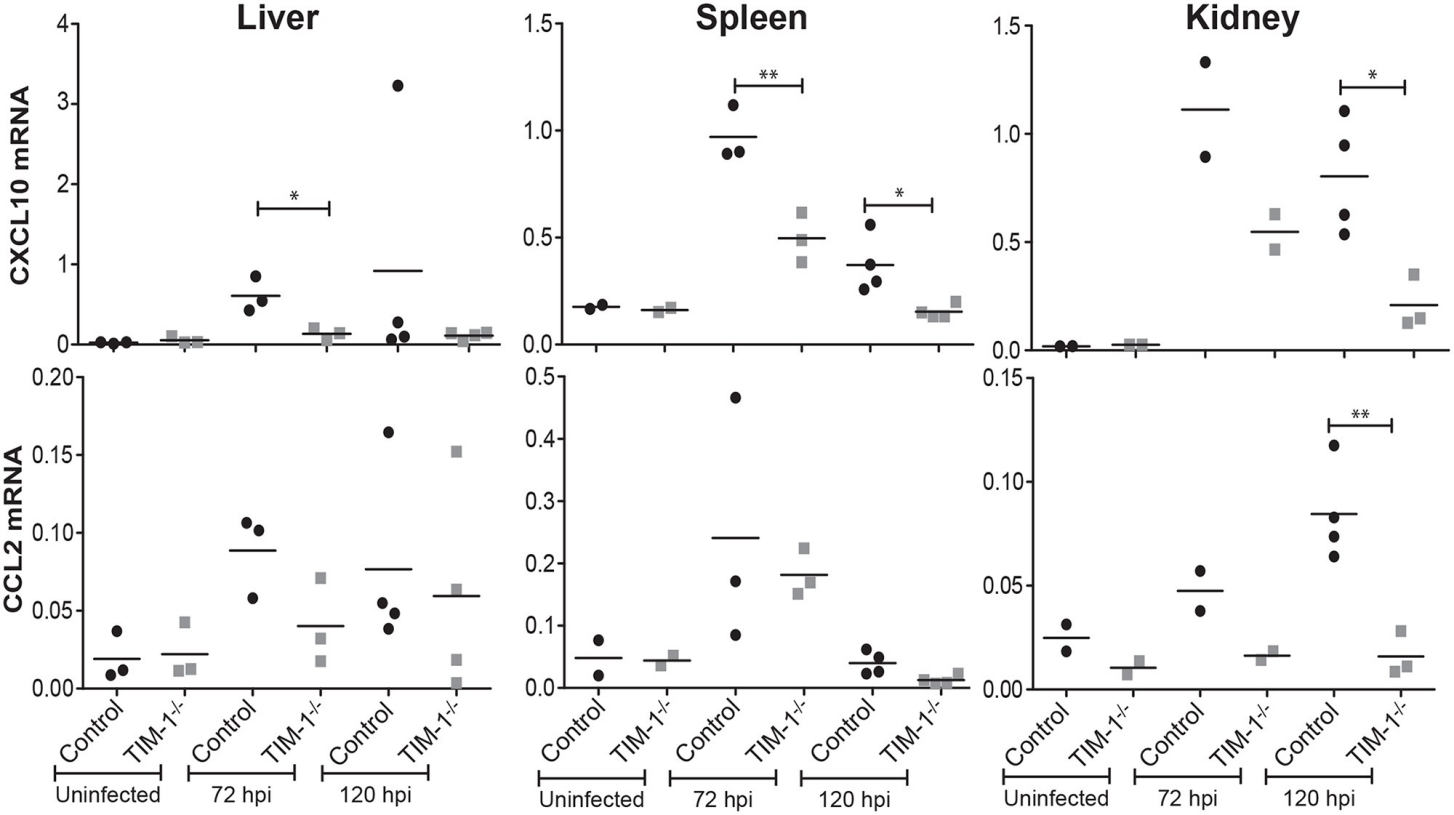
Chemokine CXCL10 and CCL2 expression in the liver, spleen and kidney of EBOV GPΔO/rVSV-infected control and TIM-1^-/-^ mice. Tissues were harvested from uninfected and infected BALB/c *Ifnar*^-/-^ (control) and BALB/c *Ifnar* ^-/-^/TIM-1^-/-^ (TIM-1^-/-^) mice. In infected mice, tissues were harvested at 3 or 5 days following infection with 10^5^ iu of EBOV GPΔO/rVSV by i.v. injection. RNA was isolated from the organs and expression of proinflammatory chemokines, mouse CXCL10 and CCL2, was quantified by qRT-PCR. Results represent chemokine expression relative to murine HPRT for at least three independent livers, spleens and kidneys. Data points represent values for individual mice. Solid lines indicate the mean for each data set. Statistical significance was determined by Student’s t-test compared the control mice for each time point and is only shown for those comparisons observed to differ. *p<0.05.

Elevated levels of several chemokines and growth factors have been implicated in fatal EBOV disease outcomes including MIP-1α, MIP-1β, MCP-1, M-CSF, MIF, IP-10, GRO-α and eotaxin [54]. Therefore, we analyzed control and TIM-1^-/-^ organs following EBOV GPΔO/rVSV infection for the chemokines, CXCL10 (IP-10) and CCL2 (MCP-1). At least one of the two transcripts for these proinflammatory chemokines in all three organs was elevated in the control mice at both day 3 and/or 5 of infection compared to the TIM-1^-/-^ mouse tissues (Fig 4). In combination with our survival and viral burden results, these observations suggest that the presence of TIM-1 in mice contributes to EBOV GP/rVSV pathogenesis through increased infection of cells in several organs at late times during infection and that this is associated with increased expression of proinflammatory chemokines.

### T cell depletion does not alter mortality associated with EBOV GP/rVSV infection

**Fig 5:**
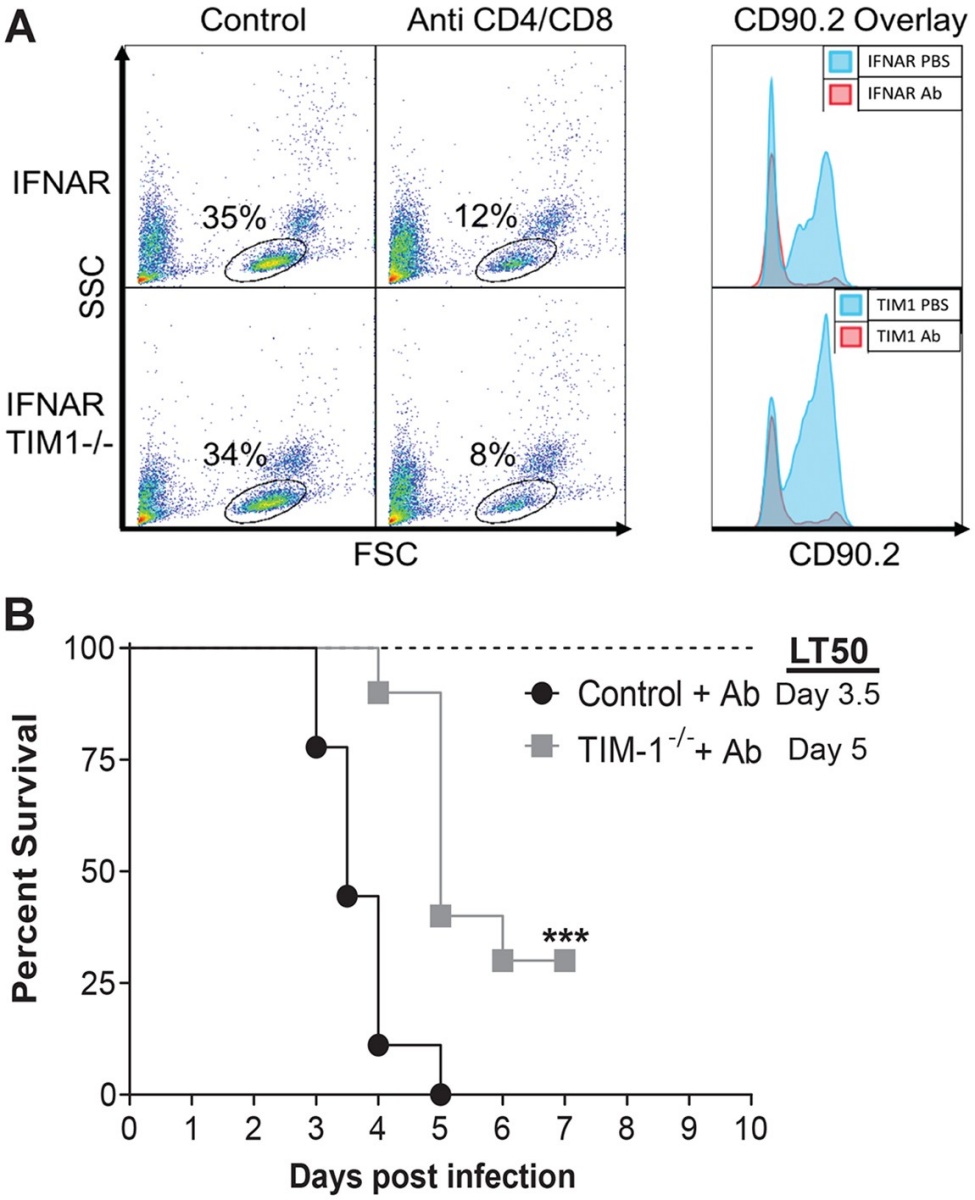
T cell depletion does not alter the survival protection conferred by the loss of TIM-1 expression. A. Intraperitoneal injection of α-CD8 mAb, clone 2.43, and α-CD4 mAb, clone GK1.5, treatment at days -1 and 2 systemically depleted T cell populations in female BALB/c *Ifnar*^-/-^ (control) and BALB/c *Ifnar*^-/-^ /TIM-1^-/-^ (TIM-1^-/-^) mice as determined by α-CD90 mAb staining of peripheral blood mononuclear cells at day 5 following EBOV GP/rVSV infection. CD90.2 overlay depicts the subset of cells gated in the panel on the left. B. Survival was assessed following infection with 7x10^2^ iu of EBOV GP/rVSV administered by intravenous infection (n = 10 mice per group) and two treatments of α-CD8 mAb and α-CD4 mAb at Day -1 and 2 from infection. Significance for survival curve was determined by Log Rank (Mantel-Cox) test. LT50 = median lethal time until death; ***p < 0.001.

TIM-1 is expressed by a number of different hematopoietic and non-hematopoietic cells [57]. Our findings indicate that virus load in spleen, an organ rich in hematopoietic cells, was not affected by the loss of TIM-1 expression, suggesting that it might be TIM-1 expression on non-hematopoietic cells late during infection that affects EBOV GP/rVSV load and survival. As others have suggested that TIM-1 on T cell subsets contribute to enhanced EBOV pathogenesis [38], we depleted T cells in control and TIM-1^-/-^ mice to assess outcomes during EBOV GP/rVSV infection. Mice were intraperitoneally administered α-CD8 mAb, 2.43, and α-CD4 mAb, GK1.5, at days -1 and 2. We verified that T cells within peripheral blood were profoundly depleted at day 5 of infection by flow cytometry following immunostaining with an α-CD90 mAb (Fig 5A). As observed for the T cell-competent mice in above studies, T cell-depleted control mice challenged with EBOV GP/rVSV succumbed to infection between 4–6 days, whereas T cell-depleted TIM-1^-/-^ mice had significantly better survival (Fig 5B). These data do not provide support for the contention that TIM-1 on T cells contributes to pathogenesis associated with our viral infection model. Instead, in total, our findings are consistent with TIM-1 expression on non-T cell populations contributing to pathogenesis.

## Discussion

Here, we show that loss of TIM-1 expression decreased overall mortality and delayed time-to-death of those mice that did succumb when challenged with EBOV GP/rVSV. The impact on survival of TIM-1 expression was similar with rVSV bearing MLD-deleted EBOV GP, indicating that the presence of the MLD did not affect the observed pathogenesis. Consistent with the enhanced survival of the TIM-1-deficient mice following virus challenge, we show that these mice also had reduced infectious virus in liver, kidney and adrenal gland at late times during infection. EBOV replication in these organs is well established and thought to contribute to overall EBOV load [47, 58–61]. The lower virus load in these organs of the TIM-1^-/-^ mice was also reflected in a ~100-fold reduction in viremia at day 5 of infection. The reduced pathology in our TIM-1-deficient mice was EBOV GP-dependent since survival associated with G/rVSV infection was unaffected by TIM-1 expression. Thus, our studies indicate that the glycoprotein present on the virions was responsible for the TIM-1-dependent changes in virus load and mouse survival.

The correlation between enhanced survival and reduced viral loads in the TIM-1^-/-^ mice suggests that TIM-1 serves as a virus receptor for EBOV in some organs. However, this role of TIM-1 must be late in infection since viremia and organ virus loads do not differ between the two mouse strains at days 1–3 of infection. A number of studies have shown at early times of infection EBOV antigens are primarily, if not exclusively, found in cells of the myeloid compartment [49, 61, 62], cells that do not express TIM-1. However, as infection progresses, additional cell types become EBOV antigen positive, suggesting a spread of virus to other cell types [50, 62]. Our data suggest that TIM-1 on some of this later group of cells contributes to virus infection and pathogenesis. Likely, late cell targets that express TIM-1 would include kidney epithelial cells [63, 64] and epithelial populations [8] in adrenal gland, eye, liver, brain and testis.

Interestingly, we did not observe that all organs previously implicated as important in EBOV infection had lower virus load in TIM-1^-/-^ mice. Splenic viral loads were high throughout infection in both control and TIM-1^-/-^ mice. These data suggested that TIM-1 expressing cells do not appreciably contribute to splenic virus loads and that splenic loads can be high in mice without those animals necessarily succumbing to infection.

While the TIM-1 does not interact directly with EBOV GP, the binding of TIM-1 to virion-associated PS has been shown to elicit viral particle entry into the endosomal compartment [9, 15] where EBOV GP is proteolytically processed, binds to NPC1 and mediates membrane fusion [17–21]. Filoviral particle entry into endosomes occurs through interactions with a number of cell surface receptors in tissue culture. However, these studies and those by Younan, et al [38] provide support that TIM-1 is important for in vivo infection and contributes to EBOV pathogenesis. Future studies to evaluate the role of additional cell surface receptors implicated in EBOV entry would provide valuable insights to the potential receptor redundancy. These receptors include other TIM family members, TAM tyrosine kinase receptors and C-type lectins.

Our studies and those performed by Younan et al. [38] delivered EBOV GP/rVSV intravenously. In other studies, we observed that intraperitoneal (i.p.) delivery of EBOV GP/rVSV or maEBOV into WT versus TIM-1^-/-^ mice was equally pathogenic. This finding may be explained by the previous observation that another TIM family member, TIM-4, is highly expressed on resident peritoneal macrophages [65] and is used as a receptor for EBOV [27]. Likely, the use of TIM-4 as a receptor within this compartment usurps the need for TIM-1 expression during i.p. challenge, even late during infection.

Surprisingly, Younan et al. did not observe that TIM-1^-/-^ mice had decreased maEBOV virema [38]. The authors reported that the genome copy number in plasma did not significantly differ in TIM-1-sufficient and -deficient mice at day 6 of infection. The discrepancy between our findings and the previous study may be due to the tissues examined, the virus administered, the quantity of virus administered and/or the timing of the sampling. One notable difference between the studies is Younan, et al. administered a very large dose of maEBOV (30,000 LD_50_) to the mice, whereas the dose of virus given to mice in our study was the minimal lethal dose determined in preliminary titration studies.

The physiological role of TIM-1 has been extensively studied. Agonistic monoclonal antibody binding to TIM-1 on CD4^+^ T, iNKT and splenic B cells induces cellular activation in a wide range of organisms from zebrafish to humans [24, 32, 33, 63, 66, 67]. This observation has led to the understanding that TIM-1 serves as a costimulatory molecule on these cells and leads to upregulation of cytokines in T and NKT cells [24, 63], as well as antibody production by B cells [67]. In contrast, transient TIM-1 expression on injured kidney epithelial cells serves an anti-inflammatory role through its uptake and clearance of apoptotic bodies [64].

Younan, et al. described the role of TIM-1 in EBOV pathology to TIM-1 stimulation of T cell cytokine and chemokine dysregulation [38]. In general, we did not observe significant differences of proinflammatory cytokines in TIM-1^+^ and TIM-1^-^ mice even at late times during infection when titer differences were notable. It is certainly possible that other cytokines, not evaluated here, might be more dramatically altered. We did observe elevated levels of the proinflammatory chemokine transcripts, CCL2 and CXCL10, in the TIM-1-sufficient mice compared to the deficient mice. We postulate that the higher levels of chemokines in TIM-1^+^ mice may reflect the innate immune responses stimulated by the higher virus load. Alternatively, as postulated by Younan, et al., the elevated chemokine profile and associated mortality in the TIM-1^+^ mice might be due to a TIM-1-dependent cytokine storm elicited by T cells [38]. We tested this latter possibility by virus challenge of T cell-depleted mice. T cell depletion did not alter EBOV GP/rVSV pathology. We found significantly greater mortality associated with virus infection of TIM-1-sufficient mice which were depleted for T cells than T cell-depleted, TIM-1-deficient mice, suggesting that T cells are not responsible for the reduced survival of TIM-1-sufficient mice. Hence, our findings do not support the conclusion that TIM-1 expression on T cells plays a significant role in the pathology associated with this acute infection.

A caveat to our studies is that our infections were performed in mice on an *Ifnar*^*-/-*^ background. It is possible that the levels of cytokines and chemokines observed in our studies are influenced by the genetic background of the mice. Others have looked at the effect of an *Ifnar*^*-/-*^ background on immune responses. Studies have shown that a number of cytokines and chemokines are suppressed by the absence of type I interferon (IFN) responses in the first 24 hours of virus infection [68, 69]. However, by 24 hours of viral infection, the burst of production of these transcripts and proteins in wild-type mice is reported to subside and levels in wild-type and *Ifnar*^*-/-*^ animals are roughly equivalent. As our studies investigate cytokine and chemokines levels at later points (days 3 and 5) during infection, the direct impact of the lack of type I IFNs may be minimal. Furthermore, not all cytokines and chemokines are suppressed in *Ifnar*^*-/-*^ mice with expression of some of these proteins enhanced by ablation of this signaling pathway [68]. In addition, during wild-type EBOV infection, it should be noted that the EBOV type I IFN antagonists, VP24 and VP35, also rapidly suppress the type I IFN pathway [70]. Thus, our EBOV GP/rVSV studies in *Ifnar*^-/-^ mice may recapitulate a number of aspects of immune suppression elicited by wild-type filoviruses.

Our results also demonstrate that TIM-1 is not important for WT VSV pathogenesis. Due to the wide cellular tropism of VSV, ubiquitous cell lipid components such as PS, phosphatidylinositol or the ganglioside GM3 originally were proposed as the VSV cell surface receptor [71–73]. However, more recent investigations have revealed that these lipids are not readily used as VSV cell surface receptors [74, 75]. Instead, the LDL receptor and its family members are proposed to serve as VSV receptors on human and mouse cells [46]. Therefore, in vivo pathogenesis induced by VSV would differ from EBOV GP/rVSV since the dependence on LDL receptors for entry is conferred by the VSV G glycoprotein [46]. Presumably the VSV membrane contains PS that can interact with TIM-1, but the affinity of VSV G for LDL receptors is likely greater than the affinity of PS in the virion envelope towards PS receptors like TIM-1. Studies from our lab have shown that only when the high affinity interactions of Lassa virus GP with its receptor, α-dystroglycan, are abrogated does TIM-1 mediate Lassa virus pseudovirion entry [76]. Future studies would be valuable to assess the ability of VSV to utilize TIM-1 as a cell surface receptor in the absence of expression of LDL receptors. A second explanation for the lack of WT VSV utilization of PS receptors, that is not mutually exclusive with the first, is that the quantity of VSV G versus EBOV glycoprotein incorporated onto the surface of VSV may be greater. While this has not been explored directly to date, if fewer EBOV glycoproteins were present on a virion, the ability of virion associated PS to interact with PS receptors might increase.

Liver and kidney dysfunction and necrosis are integral aspects of EBOV pathology of humans, NHPs [3, 77] and mice [78, 79]. Our studies indicate that TIM-1 expression is associated with elevated viral loads in the liver, kidney, adrenal gland, and brain since loss of TIM-1 significantly lowered viral burden in these organs. Future studies will need to explore the impact that TIM-1 expression has on EBOV infection of specific cells within these organs. By identifying TIM-1 expressing cells that serve as viral targets and understanding the contribution of these cells to the EBOV disease pathogenesis, we will be able to better develop TIM-1 specific therapeutics against EBOV infection.

## Supporting information

S1 Table Cytokine/Chemokine primer sequences for qRT-PCR analysis. (TIF)

S1 Fig Mortality (A, C) and weight loss (B, D) associated with increasing doses of EBOV GPΔO/rVSV (A, B) and EBOV GP/rVSV (C, D). All virus was administered iv. n = 1–3 mice per group.
(TIF)

S2 Fig Weight loss following intraperitoneal infection of *Ifnar* ^*-/-*^ mice with 10-fold serial dilutions of VSV. BALB/c *Ifnar* ^*-/-*^ mice (1–4 mice per dose) received the indicated dose of G/rVSV virus by i.p. injection. Weight loss was tracked over 10-days to determine the lowest predictably lethal dose (10^1^ infectious units). Grey lines indicate the virus doses that caused mortality in all or some of the mice over the course of the experiment with 100% of mice succumbing to the 10^1^ iu dose.
(TIF)

S3 Fig EBOV GP/rVSV serum titers. Serum was harvested from BALB/c *Ifnar*^*-/-*^ (control) or BALB/c *Ifnar*^*-/-*^ /TIM-1^-/-^ (TIM-1^-/-^) mice at days 1, 3 and 5 following infection with 10^5^ iu of EBOV GP/rVSV by i.v. injection. Titers were determined by endpoint dilution of serum on Vero cells. Solid lines indicate geometric mean for each data set. Significance was calculated by Student’s t-test comparisons of the geometric means.
(TIF)

S4 Fig Reduced viral loads in the brain but not lungs of Ifnar^-/-^/TIM-1^-/-^ mice 5 days following i.v. EBOV GP ΔO/rVSV infection. Brain (A), testis (B) and lung (C) tissue were harvested from BALB/c *Ifnar*^*-/-*^ (control) to BALB/c *Ifnar* ^*-/-*^ /TIM-1^-/-^ (TIM-1^-/-^) mice at day 5 following infection with 10^5^ iu of EBOV GP ΔO /rVSV by i.v. injection. Titers were determined by endpoint dilution of homogenized organ samples on Vero cells. Dotted line indicates the level of detection. Shown are data points for individual mice within each treatment and the bold line represents the mean titers from serum of 2–4 mice per group.
(TIF)

## References

[1] Feldmann H, Geisbert TW. Ebola haemorrhagic fever. Lancet. 2011;377(9768):849–62. 10.1016/S0140-6736(10)60667-8 21084112 PMC3406178

[2] Geisbert TW, Hensley LE, Gibb TR, Steele KE, Jaax NK, Jahrling PB. Apoptosis induced in vitro and in vivo during infection by Ebola and Marburg viruses. Lab Invest. 2000;80(2):171–86. 10701687 10.1038/labinvest.3780021

[3] Martines RB, Ng DL, Greer PW, Rollin PE, Zaki SR. Tissue and cellular tropism, pathology and pathogenesis of Ebola and Marburg viruses. J Pathol. 2015;235(2):153–74. 10.1002/path.4456 25297522

[4] Zaki SR, Goldsmith CS. Pathologic features of filovirus infections in humans. Curr Top Microbiol Immunol. 1999;235:97–116. 9893381 10.1007/978-3-642-59949-1_7

[5] Zaki SR, Shieh W-J, Greer PW, Goldsmith CS, Ferebee T, Katshitshi J, et al A novel immunohistochemical assay for the detection of Ebola virus in skin: implications for diagnosis, spread, and surveillance of Ebola hemorrhagic fever. 1999;179(Supplement 1):S36–S47. 10.1086/514319 9988163

[6] Geisbert TW, Young HA, Jahrling PB, Davis KJ, Kagan E, Hensley LE. Mechanisms underlying coagulation abnormalities in ebola hemorrhagic fever: overexpression of tissue factor in primate monocytes/macrophages is a key event. J Infect Dis. 2003;188(11):1618–29. 10.1086/379724 14639531

[7] Geisbert TW, Young HA, Jahrling PB, Davis KJ, Larsen T, Kagan E, et al Pathogenesis of Ebola hemorrhagic fever in primate models: evidence that hemorrhage is not a direct effect of virus-induced cytolysis of endothelial cells. Am J Pathol. 2003;163(6):2371–82. 10.1016/S0002-9440(10)63592-4 14633609 PMC1892396

[8] Kondratowicz AS, Lennemann NJ, Sinn PL, Davey RA, Hunt CL, Moller-Tank S, et al T-cell immunoglobulin and mucin domain 1 (TIM-1) is a receptor for Zaire Ebolavirus and Lake Victoria Marburgvirus. Proc Natl Acad Sci U S A. 2011;108(20):8426–31. 10.1073/pnas.1019030108 21536871 PMC3100998

[9] Jemielity S, Wang JJ, Chan YK, Ahmed AA, Li W, Monahan S, et al TIM-family Proteins Promote Infection of Multiple Enveloped Viruses through Virion-associated Phosphatidylserine. PLoS Pathog. 2013;9(3):e1003232 10.1371/journal.ppat.1003232 23555248 PMC3610696

[10] Alvarez CP, Lasala F, Carrillo J, Muniz O, Corbi AL, Delgado R. C-type lectins DC-SIGN and L-SIGN mediate cellular entry by Ebola virus in cis and in trans. J Virol. 2002;76(13):6841–4. 10.1128/JVI.76.13.6841-6844.2002 12050398 PMC136246

[11] Simmons G, Reeves JD, Grogan CC, Vandenberghe LH, Baribaud F, Whitbeck JC, et al DC-SIGN and DC-SIGNR bind ebola glycoproteins and enhance infection of macrophages and endothelial cells. Virology. 2003;305(1):115–23. 12504546 10.1006/viro.2002.1730

[12] Takada A, Fujioka K, Tsuiji M, Morikawa A, Higashi N, Ebihara H, et al Human macrophage C-type lectin specific for galactose and N-acetylgalactosamine promotes filovirus entry. J Virol. 2004;78(6):2943–7. 10.1128/JVI.78.6.2943-2947.2004 14990712 PMC353724

[13] Marzi A, Gramberg T, Simmons G, Moller P, Rennekamp AJ, Krumbiegel M, et al DC-SIGN and DC-SIGNR interact with the glycoprotein of Marburg virus and the S protein of severe acute respiratory syndrome coronavirus. J Virol. 2004;78(21):12090–5. 10.1128/JVI.78.21.12090-12095.2004 15479853 PMC523257

[14] Powlesland AS, Fisch T, Taylor ME, Smith DF, Tissot B, Dell A, et al A novel mechanism for LSECtin binding to Ebola virus surface glycoprotein through truncated glycans. J Biol Chem. 2008;283(1):593–602. 10.1074/jbc.M706292200 17984090 PMC2275798

[15] Moller-Tank S, Kondratowicz AS, Davey RA, Rennert PD, Maury W. Role of the phosphatidylserine receptor TIM-1 in enveloped-virus entry. J Virol. 2013;87(15):8327–41. 10.1128/JVI.01025-13 23698310 PMC3719829

[16] Mercer J, Helenius A. Vaccinia virus uses macropinocytosis and apoptotic mimicry to enter host cells. Science. 2008;320(5875):531–5. 10.1126/science.1155164 18436786

[17] Chandran K, Sullivan NJ, Felbor U, Whelan SP, Cunningham JM. Endosomal proteolysis of the Ebola virus glycoprotein is necessary for infection. Science. 2005;308(5728):1643–5. 10.1126/science.1110656 15831716 PMC4797943

[18] Carette JE, Raaben M, Wong AC, Herbert AS, Obernosterer G, Mulherkar N, et al Ebola virus entry requires the cholesterol transporter Niemann-Pick C1. Nature. 2011;477(7364):340–3. 10.1038/nature10348 21866103 PMC3175325

[19] Cote M, Misasi J, Ren T, Bruchez A, Lee K, Filone CM, et al Small molecule inhibitors reveal Niemann-Pick C1 is essential for Ebola virus infection. Nature. 2011;477(7364):344–8. 10.1038/nature10380 21866101 PMC3230319

[20] Miller EH, Obernosterer G, Raaben M, Herbert AS, Deffieu MS, Krishnan A, et al Ebola virus entry requires the host-programmed recognition of an intracellular receptor. The EMBO journal. 2012;31(8):1947–60. 10.1038/emboj.2012.53 22395071 PMC3343336

[21] Schornberg K, Matsuyama S, Kabsch K, Delos S, Bouton A, White J. Role of endosomal cathepsins in entry mediated by the Ebola virus glycoprotein. J Virol. 2006;80(8):4174–8. 10.1128/JVI.80.8.4174-4178.2006 16571833 PMC1440424

[22] Kobayashi N, Karisola P, Pena-Cruz V, Dorfman DM, Jinushi M, Umetsu SE, et al TIM-1 and TIM-4 glycoproteins bind phosphatidylserine and mediate uptake of apoptotic cells. Immunity. 2007;27(6):927–40. 10.1016/j.immuni.2007.11.011 18082433 PMC2757006

[23] Ichimura T, Asseldonk EJ, Humphreys BD, Gunaratnam L, Duffield JS, Bonventre JV. Kidney injury molecule-1 is a phosphatidylserine receptor that confers a phagocytic phenotype on epithelial cells. J Clin Invest. 2008;118(5):1657–68. 10.1172/JCI34487 18414680 PMC2293335

[24] Lee HH, Meyer EH, Goya S, Pichavant M, Kim HY, Bu X, et al Apoptotic cells activate NKT cells through T cell Ig-like mucin-like-1 resulting in airway hyperreactivity. J Immunol. 2010;185(9):5225–35. 10.4049/jimmunol.1001116 20889552 PMC3114419

[25] Kuchroo VK, Umetsu DT, DeKruyff RH, Freeman GJ. The TIM gene family: emerging roles in immunity and disease. Nat Rev Immunol. 2003;3(6):454–62. 10.1038/nri1111 12776205

[26] Meertens L, Carnec X, Lecoin MP, Ramdasi R, Guivel-Benhassine F, Lew E, et al The TIM and TAM families of phosphatidylserine receptors mediate dengue virus entry. Cell Host Microbe. 2012;12(4):544–57. 10.1016/j.chom.2012.08.009 23084921 PMC3572209

[27] Rhein BA, Brouillette RB, Schaack GA, Chiorini JA, Maury W. Characterization of Human and Murine T-Cell Immunoglobulin Mucin Domain 4 (TIM-4) IgV Domain Residues Critical for Ebola Virus Entry. J Virol. 2016;90(13):6097–111. 10.1128/JVI.00100-16 27122575 PMC4907230

[28] Morizono K, Chen IS. Role of phosphatidylserine receptors in enveloped virus infection. J Virol. 2014;88(8):4275–90. 10.1128/JVI.03287-13 24478428 PMC3993771

[29] Moller-Tank S, Albritton LM, Rennert PD, Maury W. Characterizing functional domains for TIM-mediated enveloped virus entry. J Virol. 2014;88(12):6702–13. 10.1128/JVI.00300-14 24696470 PMC4054341

[30] Santiago C, Ballesteros A, Martínez-Muñoz L, Mellado M, Kaplan GG, Freeman GJ, et al Structures of T Cell Immunoglobulin Mucin Protein 4 Show a Metal-Ion-Dependent Ligand Binding Site where Phosphatidylserine Binds. Immunity. 2007;27(6):941–51. 10.1016/j.immuni.2007.11.008 18083575 PMC2330274

[31] Binne LL, Scott ML, Rennert PD. Human TIM-1 associates with the TCR complex and up-regulates T cell activation signals. J Immunol. 2007;178(7):4342–50. 10.4049/jimmunol.178.7.4342 17371991

[32] de Souza AJ, Oak JS, Jordanhazy R, DeKruyff RH, Fruman DA, Kane LP. T cell Ig and mucin domain-1-mediated T cell activation requires recruitment and activation of phosphoinositide 3-kinase. J Immunol. 2008;180(10):6518–26. 10.4049/jimmunol.180.10.6518 18453570 PMC2637999

[33] de Souza AJ, Oriss TB, O’Malley KJ, Ray A, Kane LP. T cell Ig and mucin 1 (TIM-1) is expressed on in vivo-activated T cells and provides a costimulatory signal for T cell activation. Proceedings of the National Academy of Sciences of the United States of America. 2005;102(47):17113–8. 10.1073/pnas.0508643102 16284246 PMC1288013

[34] Bhattacharyya S, Zagorska A, Lew ED, Shrestha B, Rothlin CV, Naughton J, et al Enveloped viruses disable innate immune responses in dendritic cells by direct activation of TAM receptors. Cell Host Microbe. 2013;14(2):136–47. 10.1016/j.chom.2013.07.005 23954153 PMC3779433

[35] Bondanza A, Zimmermann VS, Rovere-Querini P, Turnay J, Dumitriu IE, Stach CM, et al Inhibition of phosphatidylserine recognition heightens the immunogenicity of irradiated lymphoma cells in vivo. J Exp Med. 2004;200(9):1157–65. 10.1084/jem.20040327 15504819 PMC2211859

[36] Hoffmann PR, Kench JA, Vondracek A, Kruk E, Daleke DL, Jordan M, et al Interaction between phosphatidylserine and the phosphatidylserine receptor inhibits immune responses in vivo. J Immunol. 2005;174(3):1393–404. 10.4049/jimmunol.174.3.1393 15661897

[37] Tan X, Jie Y, Zhang Y, Qin Y, Xu Q, Pan Z. Tim-1 blockade with RMT1-10 increases T regulatory cells and prolongs the survival of high-risk corneal allografts in mice. Exp Eye Res. 2014;122:86–93. 10.1016/j.exer.2014.02.019 24613782

[38] Younan P, Iampietro M, Nishida A, Ramanathan P, Santos RI, Dutta M, et al Ebola Virus Binding to Tim-1 on T Lymphocytes Induces a Cytokine Storm. MBio. 2017;8(5). 10.1128/mBio.00845-17 PMC5615193 28951472

[39] Curtiss ML, Gorman JV, Businga TR, Traver G, Singh M, Meyerholz DK, et al Tim-1 regulates Th2 responses in an airway hypersensitivity model. Eur J Immunol. 2012;42(3):651–61. 10.1002/eji.201141581 22144095 PMC3528103

[40] Agrawal H, Jacob N, Carreras E, Bajana S, Putterman C, Turner S, et al Deficiency of type I IFN receptor in lupus-prone New Zealand mixed 2328 mice decreases dendritic cell numbers and activation and protects from disease. J Immunol. 2009;183(9):6021–9. 10.4049/jimmunol.0803872 19812195 PMC2766036

[41] Youngner JS, Thacore HR, Kelly ME. Sensitivity of ribonucleic acid and deoxyribonucleic acid viruses to different species of interferon in cell cultures. J Virol. 1972;10(2):171–8. 4342236 10.1128/jvi.10.2.171-178.1972 PMC356447

[42] Stewart WE 2nd, Scott WD, Sulkin SE. Relative sensitivities of viruses to different species of interferon. J Virol. 1969;4(2):147–53. 4308914 10.1128/jvi.4.2.147-153.1969 PMC375849

[43] Jeffers SA, Sanders DA, Sanchez A. Covalent modifications of the ebola virus glycoprotein. J Virol. 2002;76(24):12463–72. 10.1128/JVI.76.24.12463-12472.2002 12438572 PMC136726

[44] Yang Z, Delgado R, Xu L, Todd RF, Nabel EG, Sanchez A, et al Distinct cellular interactions of secreted and transmembrane Ebola virus glycoproteins. Science. 1998;279(5353):1034–7. 10.1126/science.279.5353.1034 9461435

[45] Manicassamy B, Wang J, Jiang H, Rong L. Comprehensive analysis of ebola virus GP1 in viral entry. J Virol. 2005;79(8):4793–805. 10.1128/JVI.79.8.4793-4805.2005 15795265 PMC1069533

[46] Finkelshtein D, Werman A, Novick D, Barak S, Rubinstein M. LDL receptor and its family members serve as the cellular receptors for vesicular stomatitis virus. Proc Natl Acad Sci U S A. 2013;110(18):7306–11. 10.1073/pnas.1214441110 23589850 PMC3645523

[47] Wyers M, Formenty P, Cherel Y, Guigand L, Fernandez B, Boesch C, et al Histopathological and immunohistochemical studies of lesions associated with Ebola virus in a naturally infected chimpanzee. J Infect Dis. 1999;179 Suppl 1:S54–9. 9988165 10.1086/514300

[48] Bray M, Davis K, Geisbert T, Schmaljohn C, Huggins J. A mouse model for evaluation of prophylaxis and therapy of Ebola hemorrhagic fever. J Infect Dis. 1998;178(3):651–61. 10.1086/515386 9728532

[49] Gibb TR, Bray M, Geisbert TW, Steele KE, Kell WM, Davis KJ, et al Pathogenesis of experimental Ebola Zaire virus infection in BALB/c mice. J Comp Pathol. 2001;125(4):233–42. 10.1053/jcpa.2001.0502 11798240

[50] Hensley LE, Young HA, Jahrling PB, Geisbert TW. Proinflammatory response during Ebola virus infection of primate models: possible involvement of the tumor necrosis factor receptor superfamily. Immunol Lett. 2002;80(3):169–79. 11803049 10.1016/s0165-2478(01)00327-3

[51] Sanchez A, Lukwiya M, Bausch D, Mahanty S, Sanchez AJ, Wagoner KD, et al Analysis of human peripheral blood samples from fatal and nonfatal cases of Ebola (Sudan) hemorrhagic fever: cellular responses, virus load, and nitric oxide levels. J Virol. 2004;78(19):10370–7. 10.1128/JVI.78.19.10370-10377.2004 15367603 PMC516433

[52] Rubins KH, Hensley LE, Wahl-Jensen V, Daddario DiCaprio KM, Young HA, Reed DS, et al The temporal program of peripheral blood gene expression in the response of nonhuman primates to Ebola hemorrhagic fever. Genome Biol. 2007;8(8):R174 10.1186/gb-2007-8-8-r174 17725815 PMC2375004

[53] Hutchinson KL, Rollin PE. Cytokine and chemokine expression in humans infected with Sudan Ebola virus. J Infect Dis. 2007;196 Suppl 2:S357–63. 17940971 10.1086/520611

[54] Wauquier N, Becquart P, Padilla C, Baize S, Leroy EM. Human fatal zaire ebola virus infection is associated with an aberrant innate immunity and with massive lymphocyte apoptosis. PLoS Negl Trop Dis. 2010;4(10). 10.1371/journal.pntd.0000837 PMC2950153 20957152

[55] Martins K, Cooper C, Warren T, Wells J, Bell T, Raymond J, et al Characterization of clinical and immunological parameters during Ebola virus infection of rhesus macaques. Viral Immunol. 2015;28(1):32–41. 10.1089/vim.2014.0085 25514385

[56] Cross RW, Fenton KA, Geisbert JB, Mire CE, Geisbert TW. Modeling the Disease Course of Zaire ebolavirus Infection in the Outbred Guinea Pig. J Infect Dis. 2015;212 Suppl 2:S305–15. 26038397 10.1093/infdis/jiv237

[57] Kim HY, Chang YJ, Chuang YT, Lee HH, Kasahara DI, Martin T, et al T-cell immunoglobulin and mucin domain 1 deficiency eliminates airway hyperreactivity triggered by the recognition of airway cell death. J Allergy Clin Immunol. 2013;132(2):414–25.e6. 10.1016/j.jaci.2013.03.025 23672783 PMC3732546

[58] Ryabchikova EI, Kolesnikova LV, Luchko SV. An analysis of features of pathogenesis in two animal models of Ebola virus infection. J Infect Dis. 1999;179 Suppl 1:S199–202. 9988185 10.1086/514293

[59] Davis KJ, Anderson AO, Geisbert TW, Steele KE, Geisbert JB, Vogel P, et al Pathology of experimental Ebola virus infection in African green monkeys. Involvement of fibroblastic reticular cells. Arch Pathol Lab Med. 1997;121(8):805–19. 9278608

[60] Bird BH, Spengler JR, Chakrabarti AK, Khristova ML, Sealy TK, Coleman-McCray JD, et al Humanized Mouse Model of Ebola Virus Disease Mimics the Immune Responses in Human Disease. J Infect Dis. 2016;213(5):703–11. 10.1093/infdis/jiv538 26582961 PMC4747627

[61] Connolly BM, Steele KE, Davis KJ, Geisbert TW, Kell WM, Jaax NK, et al Pathogenesis of experimental Ebola virus infection in guinea pigs. J Infect Dis. 1999;179 Suppl 1:S203–17. 9988186 10.1086/514305

[62] Bray M, Geisbert TW. Ebola virus: the role of macrophages and dendritic cells in the pathogenesis of Ebola hemorrhagic fever. Int J Biochem Cell Biol. 2005;37(8):1560–6. 10.1016/j.biocel.2005.02.018 15896665

[63] Umetsu SE, Lee WL, McIntire JJ, Downey L, Sanjanwala B, Akbari O, et al TIM-1 induces T cell activation and inhibits the development of peripheral tolerance. Nat Immunol. 2005;6(5):447–54. 10.1038/ni1186 15793575

[64] Yang L, Brooks CR, Xiao S, Sabbisetti V, Yeung MY, Hsiao LL, et al KIM-1-mediated phagocytosis reduces acute injury to the kidney. J Clin Invest. 2015;125(4):1620–36. 10.1172/JCI75417 25751064 PMC4396492

[65] Miyanishi M, Tada K, Koike M, Uchiyama Y, Kitamura T, Nagata S. Identification of Tim4 as a phosphatidylserine receptor. Nature. 2007;450(7168):435–9. 10.1038/nature06307 17960135

[66] Xu XG, Hu JF, Ma JX, Nie L, Shao T, Xiang LX, et al Essential Roles of TIM-1 and TIM-4 Homologs in Adaptive Humoral Immunity in a Zebrafish Model. J Immunol. 2016;196(4):1686–99. 10.4049/jimmunol.1501736 26792807

[67] Ma J, Usui Y, Takeda K, Harada N, Yagita H, Okumura K, et al TIM-1 signaling in B cells regulates antibody production. Biochem Biophys Res Commun. 2011;406(2):223–8. 10.1016/j.bbrc.2011.02.021 21303660

[68] Winkelmann ER, Widman DG, Xia J, Ishikawa T, Miller-Kittrell M, Nelson MH, et al Intrinsic adjuvanting of a novel single-cycle flavivirus vaccine in the absence of type I interferon receptor signaling. Vaccine. 2012;30(8):1465–75. 10.1016/j.vaccine.2011.12.103 22226862 PMC3274573

[69] Goritzka M, Durant LR, Pereira C, Salek-Ardakani S, Openshaw PJ, Johansson C. Alpha/beta interferon receptor signaling amplifies early proinflammatory cytokine production in the lung during respiratory syncytial virus infection. J Virol. 2014;88(11):6128–36. 10.1128/JVI.00333-14 24648449 PMC4093897

[70] Basler CF. Innate immune evasion by filoviruses. Virology. 2015;479–480:122–30. 10.1016/j.virol.2015.03.030 25843618 PMC4424075

[71] Schlegel R, Tralka TS, Willingham MC, Pastan I. Inhibition of VSV binding and infectivity by phosphatidylserine: is phosphatidylserine a VSV-binding site? Cell. 1983;32(2):639–46. 6297804 10.1016/0092-8674(83)90483-x

[72] Superti F, Girmenta C, Seganti L, Orsi N. Role of sialic acid in cell receptors for vesicular stomatitis virus. Acta Virol. 1986;30(1):10–8. 2871728

[73] Mastromarino P, Conti C, Goldoni P, Hauttecoeur B, Orsi N. Characterization of membrane components of the erythrocyte involved in vesicular stomatitis virus attachment and fusion at acidic pH. J Gen Virol. 1987;68 (Pt 9):2359–69. 2821175 10.1099/0022-1317-68-9-2359

[74] Coil DA, Miller AD. Phosphatidylserine is not the cell surface receptor for vesicular stomatitis virus. J Virol. 2004;78(20):10920–6. 10.1128/JVI.78.20.10920-10926.2004 15452212 PMC521854

[75] Bloor S, Maelfait J, Krumbach R, Beyaert R, Randow F. Endoplasmic reticulum chaperone gp96 is essential for infection with vesicular stomatitis virus. Proc Natl Acad Sci U S A. 2010;107(15):6970–5. 10.1073/pnas.0908536107 20351288 PMC2872420

[76] Brouillette RB, Phillips EK, Patel R, Mahauad-Fernandez W, Moller-Tank S, Rogers KJ, et al TIM-1 Mediates Dystroglycan-Independent Entry of Lassa Virus. J Virol. 2018;92(16). 10.1128/JVI.00093-18 PMC6069209 29875238

[77] Paessler S, Walker DH. Pathogenesis of the viral hemorrhagic fevers. Annual review of pathology. 2013;8:411–40. 10.1146/annurev-pathol-020712-164041 23121052

[78] Bradfute SB, Warfield KL, Bray M. Mouse models for filovirus infections. Viruses. 2012;4(9):1477–508. 10.3390/v4091477 23170168 PMC3499815

[79] Raymond J, Bradfute S, Bray M. Filovirus infection of STAT-1 knockout mice. J Infect Dis. 2011;204 Suppl 3:S986–90. 21987780 10.1093/infdis/jir335

