# Supplementary figures and images for: TIM-1 serves as a receptor for Ebola virus in vivo, enhancing viremia and pathogenesis
Source: PLoS Negl Trop Dis. 2019 Jun 26;13(6):e0006983. doi: 10.1371/journal.pntd.0006983 (PMC6615641; doi:10.1371/journal.pntd.0006983)

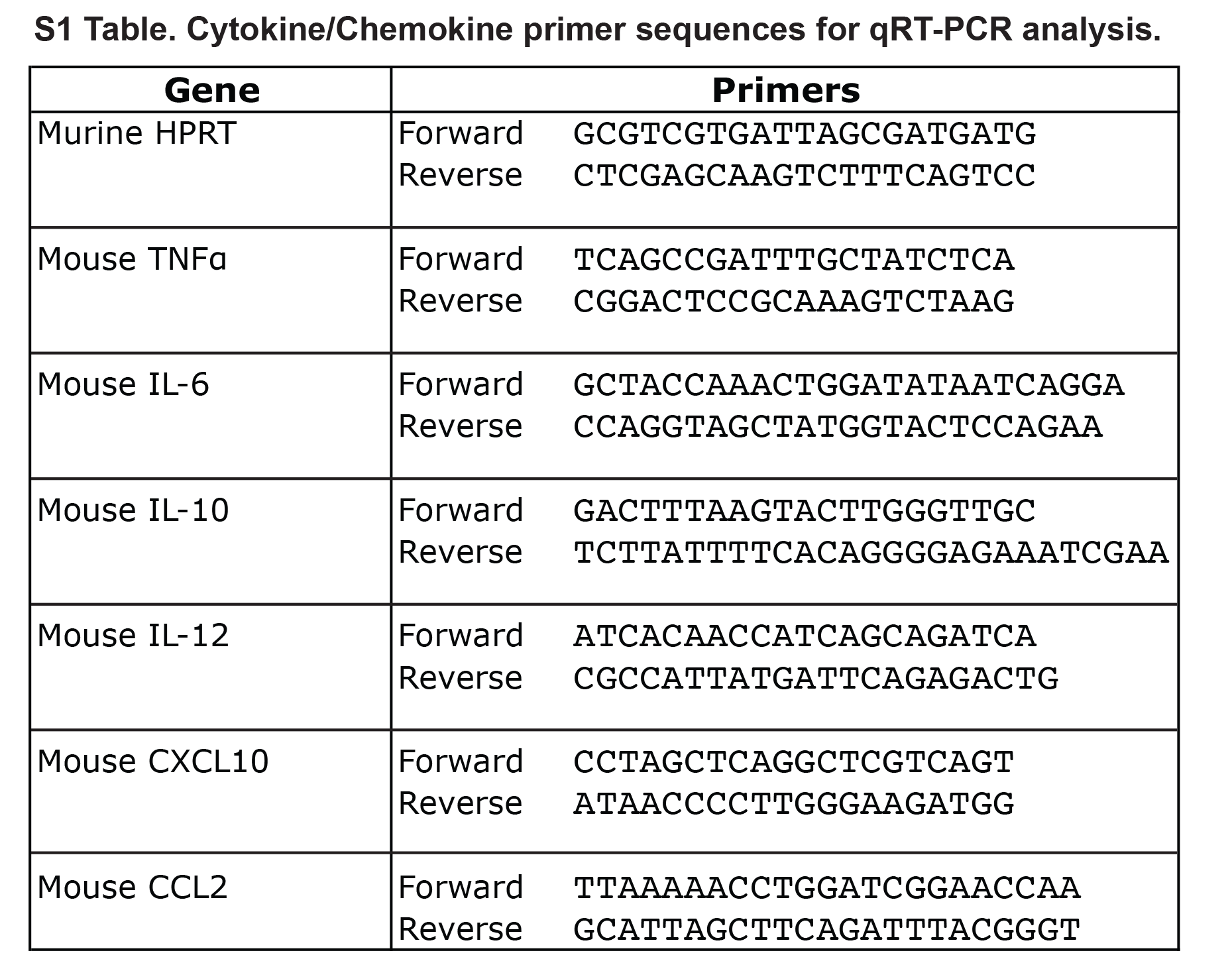

Supplement: S1 Table — (TIF) [file pntd.0006983.s001.tif]

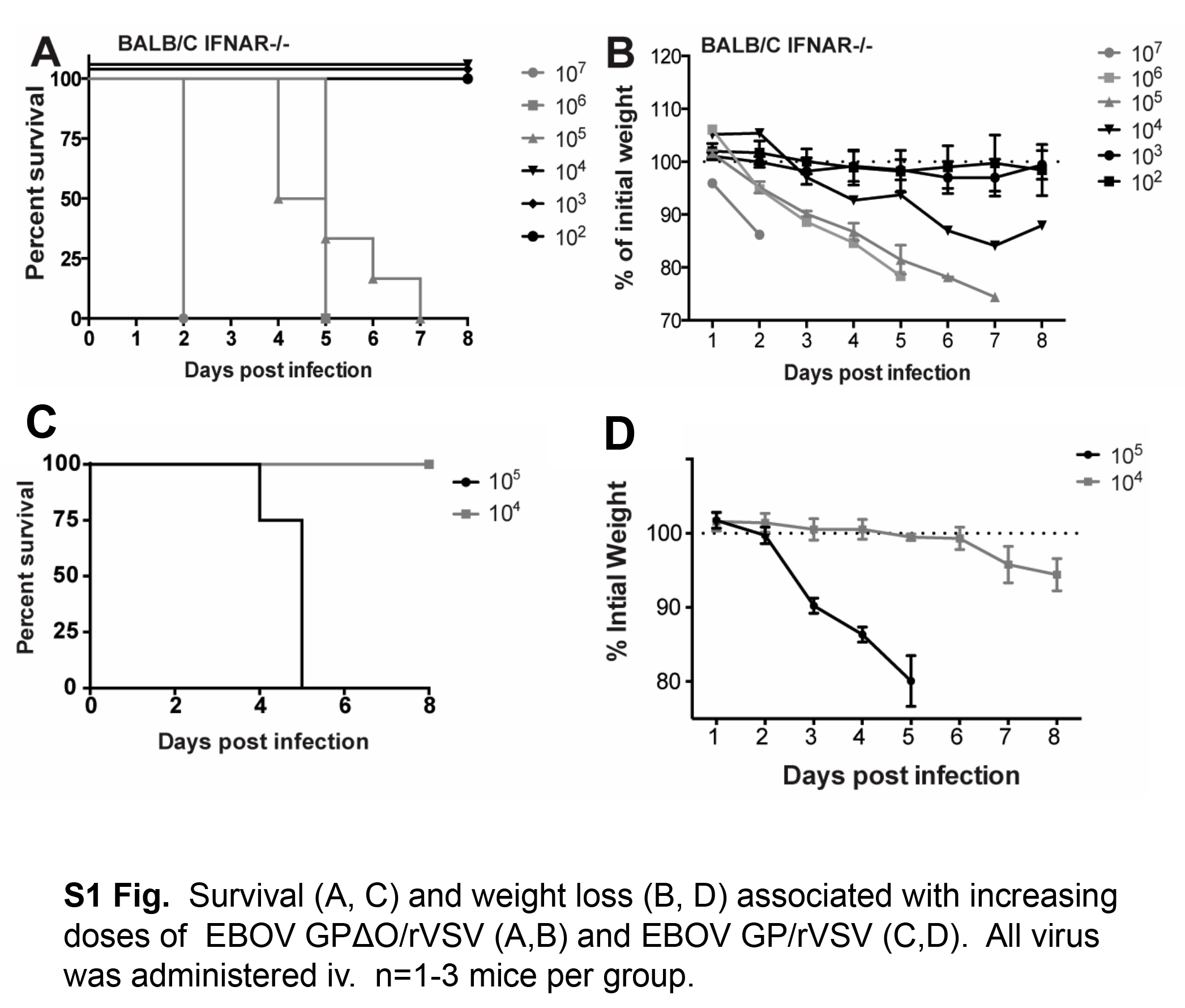

Supplement: S1 Fig — All virus was administered iv. n = 1–3 mice per group. (TIF) [file pntd.0006983.s002.tif]

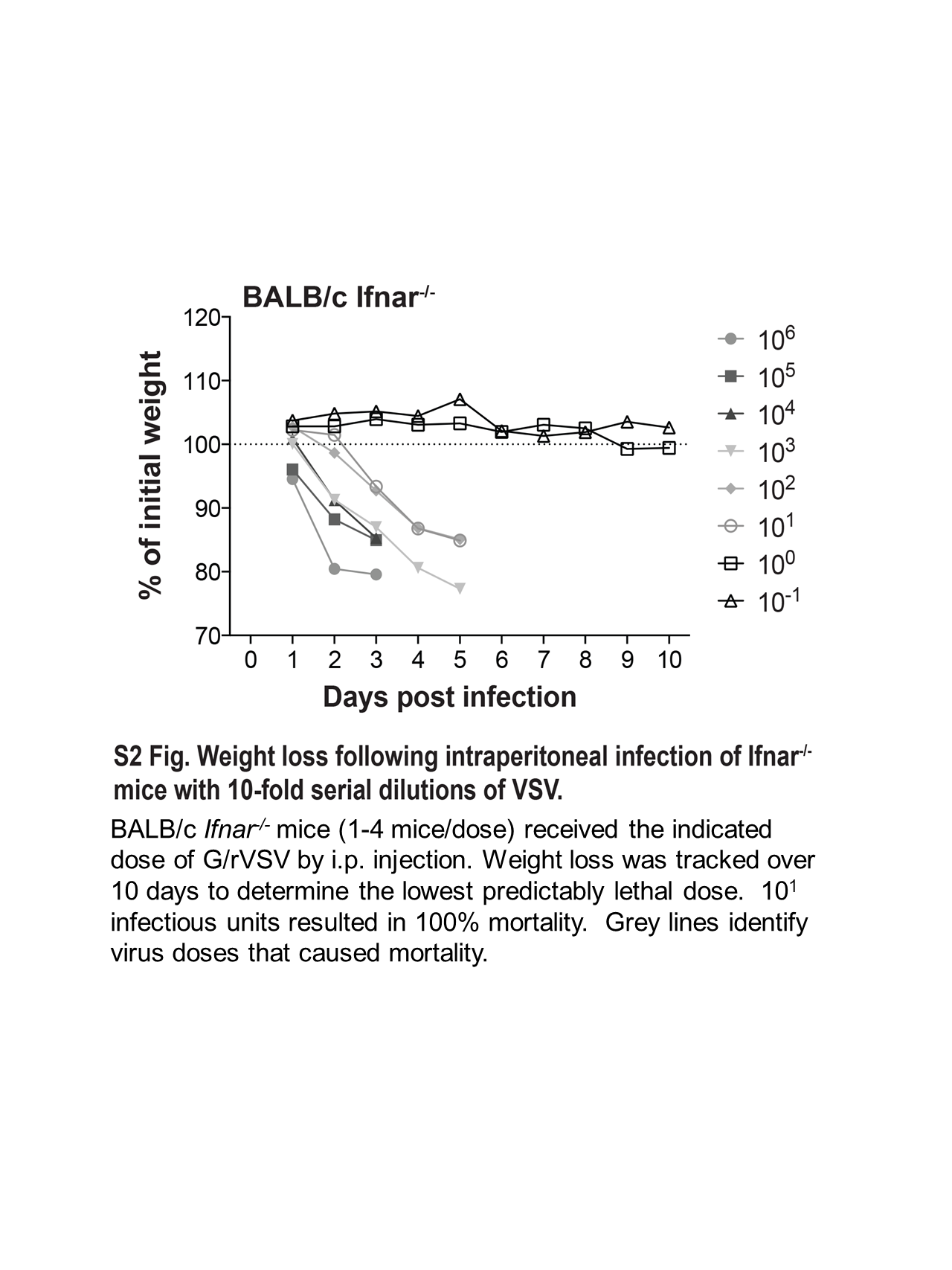

Supplement: S2 Fig — BALB/c Ifnar -/- mice (1–4 mice per dose) received the indicated dose of G/rVSV virus by i.p. injection. Weight loss was tracked over 10-days to determine the lowest predictably lethal dose (101 infectious units). Grey lines indicate the virus doses that caused mortality in all or some of the mice over the course of the experiment with 100% of mice succumbing to the 101 iu dose. (TIF) [file pntd.0006983.s003.tif]

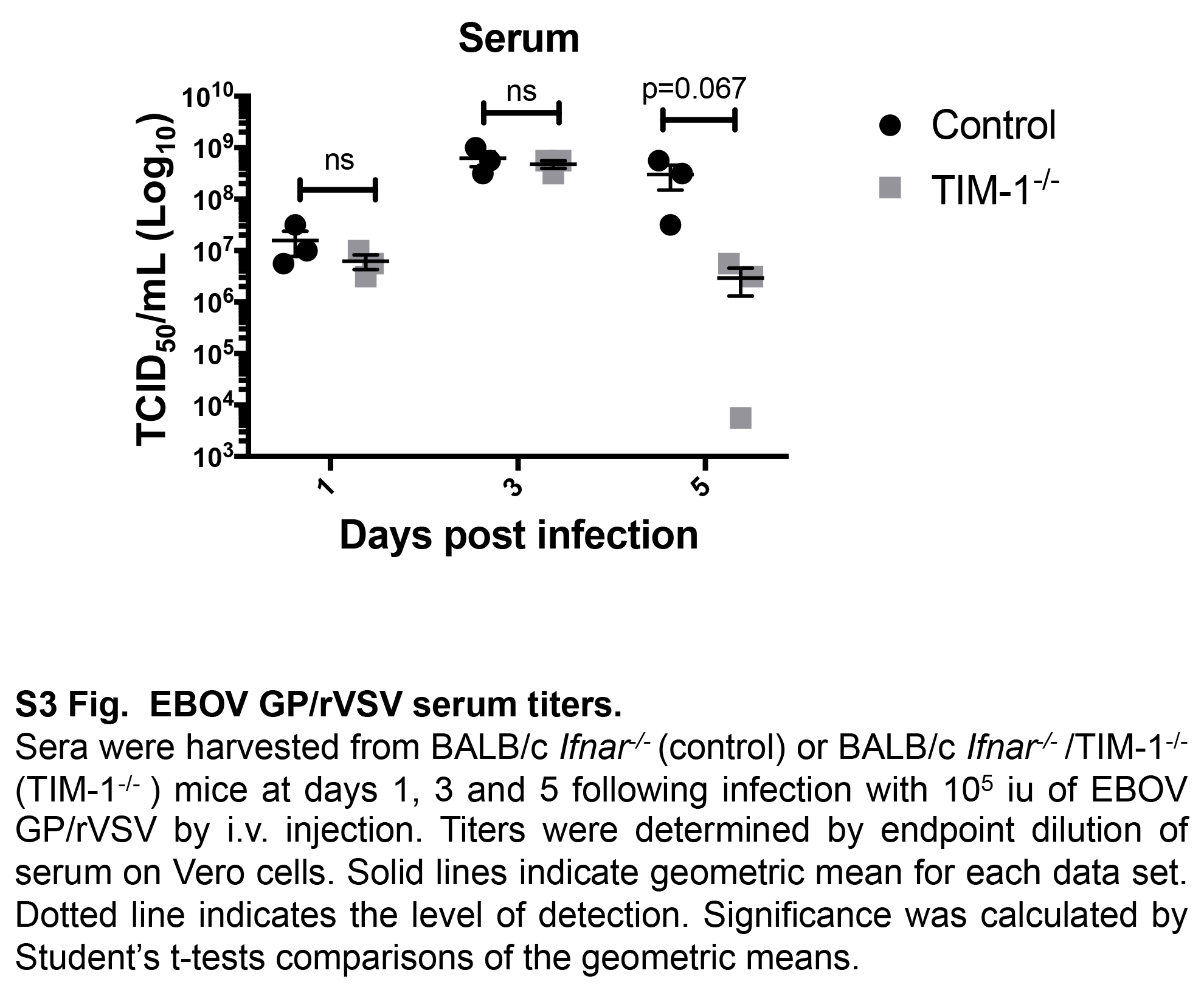

Supplement: S3 Fig — Serum was harvested from BALB/c Ifnar-/- (control) or BALB/c Ifnar-/- /TIM-1-/- (TIM-1-/-) mice at days 1, 3 and 5 following infection with 105 iu of EBOV GP/rVSV by i.v. injection. Titers were determined by endpoint dilution of serum on Vero cells. Solid lines indicate geometric mean for each data set. Significance was calculated by Student’s t-test comparisons of the geometric means. (TIF) [file pntd.0006983.s004.tif]

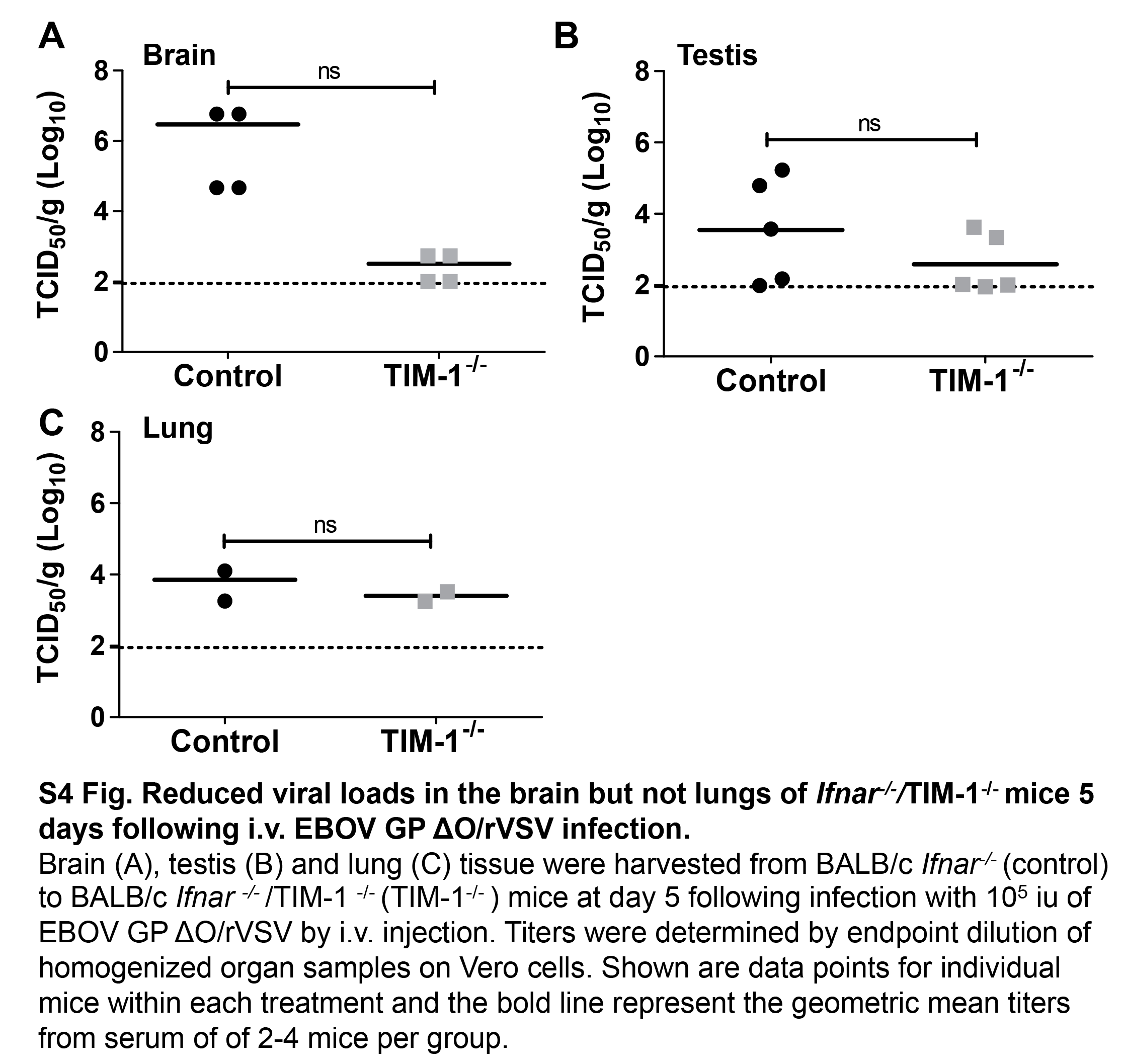

Supplement: S4 Fig — Brain (A), testis (B) and lung (C) tissue were harvested from BALB/c Ifnar-/- (control) to BALB/c Ifnar -/- /TIM-1-/- (TIM-1-/-) mice at day 5 following infection with 105 iu of EBOV GP ΔO /rVSV by i.v. injection. Titers were determined by endpoint dilution of homogenized organ samples on Vero cells. Dotted line indicates the level of detection. Shown are data points for individual mice within each treatment and the bold line represents the mean titers from serum of 2–4 mice per group. (TIF) [file pntd.0006983.s005.tif]
